# Supplementary material for: Immunogenetic losses co-occurred with seahorse male pregnancy and mutation in tlx1 accompanied functional asplenia
Source: Nat Commun. 2022 Dec 9;13:7610. doi: 10.1038/s41467-022-35338-7 (PMC9734139; doi:10.1038/s41467-022-35338-7)
Supplement: Supplementary file 1 — Supplementary Information [file 41467_2022_35338_MOESM1_ESM.pdf]

Supplementary Information to:

Immunogenetic losses co-occurred with seahorse male pregnancy and mutation in *tlx1* accompanied functional asplenia

Yali Liu[1,2,3]†, Meng Qu[1,2]†, Han Jiang[1,3]†, Ralf Schneider[4]†, Geng Qin[1,2], Wei Luo[1], Haiyan Yu[1], Bo Zhang[1], Xin Wang[1,2], Yanhong Zhang[1,2], Huixian Zhang[1,2], Zhixin Zhang[1,5], Yongli Wu[1], Yingyi Zhang[1,3], Jianping Yin[1,2], Si Zhang[1,2], Byrappa Venkatesh[6], Olivia Roth[4]*, Axel Meyer[7]*, Qiang Lin[1,2,3]*

†Authors contributed equally to this work.

*Correspondence to: linqiang@scsio.ac.cn (Q.L.); axel.meyer@uni-konstanz.de (A.M.); oroth@zoologie.uni-kiel.de

[1]CAS Key Laboratory of Tropical Marine Bio-Resources and Ecology, South China Sea Institute of Oceanology, Chinese Academy of Sciences, 510301 Guangzhou, China.

[2]Guangdong Provincial Key Laboratory of Applied Marine Biology, South China Sea Institute of Oceanology, Chinese Academy of Sciences, Guangzhou 510301, PR China.

[3]University of Chinese Academy of Sciences, 100101 Beijing, China.

[4]Marine Evolutionary Ecology, Zoological Institute, Kiel University, 24118 Kiel, Germany.

[5]Graduate School of Marine Science and Technology, Tokyo University of Marine Science and Technology, Minato, Tokyo, Japan.

[6]Institute of Molecular and Cell Biology, A*STAR, 138673 Biopolis, Singapore.

[7]Department of Biology, University of Konstanz, 78464 Konstanz, Germany.

Table of Contents

Supplementary Notes **Page 3-12**

Supplementary Figures 1-30 **Page 13-44**

Supplementary Tables 1-18 **Page 45-71**

Supplementary Reference 1-25 **Page 72-74**

Supplementary Notes

1. Genome assembly and annotation

We calculated and plotted the 19-mer depth distribution of *H. mohnikei* and *H. zosterae*, and the results are presented in **Supplementary Figure 1** and **Supplementary Table 1-3**. In the assembly step, the assembly statistics are shown in **Supplementary Table 4-5**. Benchmarking Universal Single-Copy Orthologs (BUSCOs) assessment showed that our assembly captured 93.46% and 92.95% of complete BUSCOs of *H. mohnikei* and *H. zosterae*, respectively. For Hi-C sequencing, the sequence interaction matrices are shown in **Supplementary Figure 2**, and the statistical analysis results of chromosome assembles are summarized in **Supplementary Table 6**. Furthermore, the pseudo-chromosome syntenic relationship between *H. zosterae* and the lined seahorse *H. erectus* (2n = 22) was analyzed. The result revealed that the syntenic relationship between these two seahorses showed few ordering discrepancies and significant synteny between chromosomes (**Supplementary Figure 3)**.

The results of genome annotation are summarized in **Supplementary Table 7-8**. Finally, we obtained a total of 21,386 and 21,605 genes from the *H. mohnikei* and *H. zosterae* genomes, respectively (**Supplementary Table 9**). In total, 95.43% *(H. mohnikei)* and 96.09% *(H. zosterae)* of the predicted genes were annotated using the different databases (**Supplementary Table 10-11**). For annotation of non-coding RNAs (ncRNAs), microRNAs (miRNA) and rRNAs were predicted using Infernal with Rfam [1], whereas tRNAs were screened using tRNAscan-SE [2]. For pseudogene prediction, GenBlastA (v1.0.4) [3] was applied to identify the candidate pseudogene by homologous searching against genome data. Next, GeneWise (v2.4.1) [4] was used to search for immature termination and frameshift mutation of the pseudogenes (**Supplementary Table 12**).

2. Phylogenetic analysis

To determine the phylogenetic position of *H. mohnikei* and *H. zosterae*, a phylogenetic analysis was conducted using a whole-genome protein datasets of four

Hippocampus spp. and nine other representative ray-finned fishes. The resultant tree shows maximal bootstrap support for all nodes. Our results showed that the phylogenetic relationships of these orders in this tree (**Supplementary Figure 4a**) were consistent with a recent comprehensive phylogenetic study based on sequences from 303 ray-finned fish species representing 66 orders [5].

3. Comparative genomic analysis

Expansion and contraction of gene families

Gene expansion and contraction results for each branch of the phylogenetic tree were estimated. At last, four seahorses were found to possess 163 significantly expanded and 1260 significantly contracted gene families (**Supplementary Figure 4a and Supplementary Data 1**). GO and KEGG enrichment analysis of contracted gene families in seahorses were conducted by the GOseq R package and KOBAS software [6], respectively. GO enrichment analysis showed that the contracted gene families were related to inflammasome complex and virus receptor activity (**Supplementary Figure 5a**). The KEGG pathway-based analysis suggested that the contracted gene families were related to several important immune-related pathways, including autoimmune thyroid disease, allograft rejection, graft-versus-host disease, herpes simplex infection, and antigen processing and presentation pathways (**Fig. 1c)**. The full lists of significantly enriched pathways are shown in **Supplementary Table 13 and Supplementary Data 2**.

Gene loss

Similar to gene contraction analysis, KEGG enrichment analysis showed that the gene losses were related to the allograft rejection, graft-versus-host disease pathway and antigen processing and presentation (**Supplementary Figure 4b and Supplementary Data 3**). These gene losses, contracted gene families, and enriched pathways, together with the loss of vital immune organs (the spleen and GALT), implied that these *Hippocampus* fishes might harbor unique immune-genetic characteristics, which may be the reason for the modifications in the seahorse immune system.

Positive selection of genes

A total of 103 PSGs were identified in four seahorses (*FDR* < 0.05) (**Supplementary Data 4**). Notably, among these genes, we found several genes that play important roles in the response of the immune system, including *pleckstrin homology-like domain family B member 1* (*phldb1*), *legumain (lgmn)* and *B-cell CLL/lymphoma 6 member B protein (bcl6b)*.

Rapidly evolving genes

In total, 693 REGs exhibiting signatures indicating selection were identified in the seahorses. The REGs are listed in **Supplementary Data 5**. GO and KEGG analyses revealed that these REGs were significantly enriched in the cell cycle, proteasome and neuroactive ligand-receptor interaction pathway (**Supplementary Figure 5b, Supplementary Figure 6a, Supplementary Data 6, and Supplementary Table 14**).

Lineage-specific mutated genes

We obtained a total of 845 LSGs including 1158 seahorse lineage-specific mutated sites. The LSGs are listed in **Supplementary Data 7**. The primordium of the spleen originates from local mesenchymal cells, which are derived from the mesoderm. Previous studies in mice and zebrafish revealed that spleen development is related to several homeobox transcription factors expressed in the spleen primordium, including Pre-B-cell leukemia transcription factor 1 (Pbx1), T Cell Leukemia Homeobox 1 (Tlx1), NK3 Homeobox 2 (Bapx1), SRY-box transcription factor 11 (Sox11), Transcription Factor 21 (Tcf21), and NK2 Homeobox 5 (Nkx2.5) [7-9]. Among these, Tlx1, which was reported to control splenic primordia cell fate specification and organ expansion, is the only one in which asplenia is not accompanied by other developmental deficiencies [10,11]. Coincidently, among these LSGs, Tlx1 (877-879, Ala to Thr) showed lineage-specific mutation in the seahorses. Thus, we speculate that *tlx1* might be a vital candidate gene of asplenia in seahorse.

GO and KEGG enrichment analyses of LSGs were performed, and the results are presented in **Supplementary Data 8 and Supplementary 15**. These lineage-specific mutated genes were also significantly enriched in the ubiquitin mediated proteolysis,

spliceosome, glycosylphosphatidylinositol (GPI)-anchor biosynthesis and mTOR signaling pathway (**Supplementary Figure 6b**).

4. Validation of the specific mutation of *tlx1* in seahorses

Local gene synteny and phylogenetic analysis of the Tlx gene families of lined seahorse

Tlx1, once had been mistaken for Hox11, belongs to T cell leukemia homeobox gene families (which include Tlx1, Tlx2, and Tlx3) and contains a well-conserved DNA sequence known as the “homeobox”. Our local gene synteny and phylogenetic analysis showed that lined seahorse had intact *tlx* gene family members, including one copy of *tlx1*, *tlx2*, and *tlx3* each (**Supplementary Figure 7**).

Cloning and structure prediction of the tlx1 ORF in lined seahorse

PCR analysis was conducted for *tlx1* to verify the lineage-specific mutation of *tlx1*. Because the fresh samples of seahorses were limited, we used the best available fresh samples of lined seahorse for RNA isolation from a seahorse farm in Zhangzhou, Fujian, China. The primers are listed as follow: forward primer (F): 5′-ATGGATCACATGGGACTAGC-3′; reverse primer (R): 5′-CTACTCGCAGGCCGTAACG-3′). The thermocycling conditions were conducted based on a standard PCR procedure. The PCR products were loaded on a 1.5% agarose gel and electrophoresed at 130 V for 25 min. The results were photographed under a UV light. The 3D structures of the Tlx1 protein in lined seahorse were predicted using I-TASSER. Our results revealed that the *tlx1* ORF in lined seahorse contained an Ala to Thr mutation in a representative homeobox domain, which is consistent with the genome sequencing results (**Supplementary Figure 8, 9**). Meanwhile, we obtained good C-scores (-4.03) and TM-scores (0.28±0.09), indicating the correctness of the structures. C-score is a confidence score for estimating the quality of predicted models. TM-score is a recently proposed scale for measuring the similarity between two structures (**Supplementary Figure 10**).

Validation of Tlx1 amino acids in 18 seahorse species and other Syngnathidae fishes

The PCR and sequencing results showed that all the seahorses exhibited an Ala to Thr mutation (**Supplementary Figure 11a, Supplementary Figure 12-13**), indicating that the fixation might have occurred before seahorse speciation. We also investigated the mutation locus by comparison with the genomes of all other available members of the Syngnathinae and Nerophinae subfamily. The results showed that all investigated members of the genus *Syngnathus* (all with quite derived, closed brooding organs, and are closely related to seahorses) also shared the described mutation, whereas the less closely related fishes (which have less derived brooding organs), such as seadragon or alligator pipefish, retained the ancestral Ala in this locus (**Supplementary Figure 11b**). Seadragon and alligator pipefish have open brood patch. Meanwhile, *Oostethus manadensis* and *Nerophis ophidion* show the amino acid substitutions of A to L and A to I, respectively (**Supplementary Figure 11b**). As mutations in the exons of protein-coding genes can lead to substantial phenotypic changes, we hypothesized that the identified mutation in the conserved homeobox domain of *tlx1* might be associated with the loss of the spleen in seahorses.

5. Establishment of *tlx1* knockout and point mutation zebrafish lines by CRISPR/Cas9-based genome editing

Generation of tlx1$^{▲}$ *zebrafish lines*

To explore the function of *tlx1* in spleen development, we generated a *tlx1* knockout zebrafish line (*tlx1*$^{▲}$) (**Fig. 3a and Supplementary Figure 15**). Finally, we generated two *tlx1* knockout zebrafish lines. Two *tlx1* nonsense alleles with 122-bp deletion *tlx1*$^{▲}$ (-122) and 5-bp deletion *tlx1*$^{▲}$ (-5) in the first exon were generated (the red arrowheads and the yellow-backed sequence), which caused frame-shift mutations at the position 43 and 47 AA, respectively (the red-backed sequence), as well as premature transcription termination event at the position 47 and 58 AA (the green-backed sequence). Considering that the phenotypic consistency of different mutant lines of the same gene was confirmed in subsequent experiments, the wild-type and homozygous mutant lines of *tlx1* are referred to as WT and *tlx1*$^{▲}$, respectively, unless

otherwise stated. The sequences of the wild type and *tlx1*$^{\blacktriangle}$ fishes were listed in **Supplementary Data 10**.

Generation of tlx1^{A208T} *and tlx1*^{A207T} *zebrafish lines*

To investigate whether the phenotypic consequences in the seahorses were caused by a missense mutation in *tlx1*, we generated a *tlx1* point mutation zebrafish line (*tlx1*^{A208T}) by CRISPR/Cas9-mediated homologous recombination (HR). In addition, another A to T point-mutation was found at the adjacent site of the seahorse-specific mutation, and the zebrafish homozygote line (*tlx1*^{A207T}) was produced (**Fig. 3a and Supplementary Figure 15**). The primers were listed in **Supplementary Data 10**.

6. Whole mount *in situ* hybridization of *tlx1*

The expression of the *tlx1* gene was examined *in situ* in lined seahorse embryos. We found positive signals for *tlx1* in the hindbrain and pharyngeal arches in the early- and mid-stage embryos, but not in the late-stage embryos. Furthermore, unlike mice and zebrafish [9,12], seahorse did not show *tlx1* expression in the corresponding splenic primordium region during embryogenesis (**Supplementary Figure 19a**).

7. Transcriptome analyses of wild-type, *tlx1*$^{\blacktriangle}$, and *tlx1*^{A208T} zebrafishes

Several studies have shown that as a transcription factor, *tlx1* is not only a critical regulatory element for the spleen primordium development but also expressed in the brain and plays a role in the differentiation and/or survival of specific neuronal populations [13]. Our results revealed that compared to the *tlx1*$^{\blacktriangle}$ line, transcriptomic patterns of the *tlx1*^{A208T} line had more in common with those of the wild types, with a lower number of DEGs in the brain, kidney, liver, and intestine (896/303, 506/242, 841/270, and 834/412, respectively) (**Fig. 3d-e. Supplementary Figure 19b-h, Supplementary Data 11**), indicating that their differences were caused by full gene knockout and point mutation. Further GO analyses between *tlx1*$^{\blacktriangle}$ and *tlx1*^{A208T} lines revealed that the DEGs in the brain were enriched in the membrane component, oxidase enzyme activity, and biosynthetic processes; DEGs in the kidney were enriched in the integral component of membrane and G-protein coupled receptor signaling pathway; DEGs in the liver were enriched in immune response and

leukocyte migration; and DEGs in the intestine were enriched in the regulation of acrosome reaction, cell-cell recognition, and acrosin binding (**Fig. 3f, Supplementary Figure 20, Supplementary Data 12**). Our results suggested that although *tlx1*▲ and *tlx1*^{A208T} zebrafishes had the same asplenia phenotype, the effects might differ in different organs.

8. Comparative whole genome search for seahorse immune/pregnancy genes

Detailed evaluation for the regressive evolution of the immune system was necessary for the further investigation of "male-pregnancy" evolution. We found the complete loss or reduced number or noticeable changes in several immune-related genes in four seahorse species and six other Syngnathidae species compared to other vertebrates: (1) loss of the *Basic leucine zipper transcriptional factor ATF-like 3 (batf3)* gene; (2) varied diversity of major histocompatibility complex (MHC) genes; (3) loss of the *T-cell surface glycoprotein cd5 (cd5)* gene; (4) Variation in the B cell receptor complex (*CD79a/b*); (5) reduced number of variable regions in *immunoglobulin heavy chain* (*ighv*); (6) loss of the *Complement C4 (C4)* gene; (7) loss of the *Forkhead box protein P3 (foxp3)*gene (**Fig. 4a**). The GenBank accession numbers of these gene families used for phylogenetic analysis are listed in **Supplementary Data 13**.

<u>*Loss of batf3*</u>

Lymphocytes are reported to be involved in the allorecognition and initiation of allograft rejection, which is necessary for establishing, maintaining, and completing a healthy pregnancy. As a member of the antigen-presenting cell (APC) family, dendritic cells present antigens T cells to promote immunity against foreigner materials and self-tolerance. *batf3* is a crucial transcription factor for the development of CD8-alpha+ classical dendritic cells; *batf3*-/- mice lack specific splenic CD8α^+ dendritic cell subset and have a defect in cross-presentation. Interestingly, we observed a typical loss of *batf3* in the genomes of all *Syngnathidae* species compared to other representative vertebrates (**Supplementary Figure 21a and Supplementary Figure 22**).

Varied diversity of MHC pathway molecules

The general organization of the MHC genes is vital for the allorecognition and histocompatibility in jawed vertebrates. MHC I and MHC II vary greatly, and are involved in the recognition of non-self peptides and their presentation to $CD8^+$ and $CD4^+$ T cells, respectively. Our results revealed that MHC I are ubiquitous in Syngnathidae, whereas MHC II are lost in *Syngnathus* species. These results are similar to those of a previous study by Roth et al. 2020. Moreover, we found that the *cd8b* gene, which is responsible for encoding the heterodimeric CD8αβ and activating $CD8^+$ T lymphocytes, was absent in *Hippocampu*s and *Syngnathus* species (**Fig. 4a**). Similarly, a comparative genomic study of Syngnathidae also revealed that the *cd8b* gene was lost in the other two seahorses (*H. kuda* and *Hippocampus whitei*).

Loss of cd5

Previous studies reported that the lack of B1-a lymphocytes after splenectomy suggests that the spleen is essential for producing B-1a B cells [15]. These B lymphocytes play a vital role in producing natural antibodies (mainly IgM) and resisting pathogen infection [16]. Our results showed that the *cd5* gene, which encodes the surface molecule of B-1a B cells, was lost in the genomes of all four species (**Supplementary Figure 21b and Supplementary Figure 23**), indicating that the absence of the spleen might co-evolved with the significant changes in B lymphocytes and the natural protective antibody.

Variation in the B cell receptor complex (CD79a/b)

Antibody-mediated allograft rejection is essential in the process of transplantation. We therefore examined genes that essential for the humoral arm of the adaptive immunity of the B cell receptor complex, comprising a CD79 a/b heterodimer and immunoglobulins. Upon the binding of the B cell receptor to self/non-self peptides, CD79a/b initiates a signaling cascade response and encompasses protein tyrosine phosphorylation with its immunoreceptor tyrosine-based activation motifs (ITAMs). Both CD79 chains contain an ITAM in their intracellular tails, which they use to propagate a signal in a B cell, and it is difficult to maintain the homeostasis of B cells without the functional signaling subunits of

ITAMs [17]. However, we found that one ITAM in the *cd79a* gene was missing in the four seahorse species (**Supplementary Figure 24-25**). As a vital element of the B cell receptor complex, structural variations in the *cd79a* gene might reduce the functional signaling ability of B cell receptors.

Reduced number of ighvs

Immunoglobulins consist of two heavy (H) and two light (L) chains and assume a fundamental role in adaptive immunity by unequivocally restricting antigens. They acquire the ability to create diverse receptors through somatic rearrangement of variable (V), joining (J), and in some cases, diversity (D) gene segments, namely V(D)J recombination [16]. Herein, after searching the whole-genome of four species, we found that the number of *ighv* in seahorses was significantly less (3~6) than that in other teleosts (≥35) (**Fig. 4a and Supplementary Figure 26a**). Gene microsynteny of the immunoglobulin heavy chain loci also showed significantly reduced scaffold length of *ighv* in four seahorses (**Supplementary Figure 26b**). Furthermore, the other six syngnathid species also harbored relatively small numbers of *ighv* (**Fig. 4a**). Previous studies suggested that serum immunoglobulin (including IgG, IgM, and IgA) varies during gestation, which implies an important role of immunoglobulins in mediating the maternal-tolerated homograft [18]. Taken together, these modifications in antibody-mediated immunity might contribute to the trade-offs for a successful pregnancy in seahorses and other syngnathids.

Impairment of the complement system in Syngnathidae

The classical pathway of the complement system in vertebrates can be triggered by antibody-antigen complexes binding to C1, followed by the activation of C2 and C4 and the formation of the C3 convertase, C4b2a, which splits C3 into C3a and C3b, causing a cascade of further cleavage and activation events. Previous studies reported that antibodies can acutely induce rejection through the fixation of the complement system, especially the complement component 4d (C4d), a durable *in situ* marker of complement activation [19]. A phylogenetic tree of C3-C5 was generated, which conforms the C3-C5 gene sequence we found in seahorses are correct, however, C4 gene was absent in all detected seahorse species that examined in this study

(**Supplementary Figure 21c and Supplementary Figure 28a**). To verify this result, vista plot was generated (**Supplementary Figure 27**), and also revealed the loss of C4 in other Syngnathids. Among C1-C9 genes, the key components C3, C4 and C5 belong to the same family [20,21]. In addition, we also counted the copy number of C3 and C5 in vertebrates (**Fig. 4a**). We found that C3 gene duplicated in all fish species except coelacanth with the copy number up to eight (zebrafish), while the syngnathids have the lowest copy number of only two (**Fig. 4a**).

foxp gene family in Syngnathidae

Regulatory T (Treg) cells have been suggested to maintain self-immunotolerance by suppressing inflammation and play an essential role during pregnancy in mammals [22,23]. *foxp3* acts as a crucial regulator for the establishment and maintenance of Treg cells. We also searched the *foxp3* gene in the four seahorses, but did not find the gene in any of the species. To verify this result, we generated a phylogenetic tree of all *foxp* genes we found in seahorses and other representative vertebrates using the amino acid sequences. Our results supported that the *foxp1*, *foxp2,* and *foxp4* genes we identified in four seahorses were correct, and that the *foxp3* gene was missing in all studied seahorses (**Supplementary Figure 28b**). We also found that the *foxp3* gene was missing in six other Syngnathidae species. Previous studies have found that patients with splenectomy showed decreased blood FOXP3^{+} Treg cells, which is similar to our results. During pregnancy, immune tolerance is enhanced by an increase in Treg cells, which ensures the successful implantation of embryos. The loss of *foxp3* indicates that the seahorses might adopt their own unique immune tolerance mechanism compared with that of mammals.

Comparative genomic search for these genes in relatively closely Syngnathiformes

We also have looked at the presence/absence of the immune-related genes including *batf3*, *C3*, *C4* and *foxp3* that are missing in Sygnathids in relatively closely related other Syngnathiformes genomes (*Aeoliscus strigatus* and *Fistularia tabacaria*). Our results showed that these four gene are intact in both species. The scaffold location and sequences were listed in **Supplementary Data 14.**

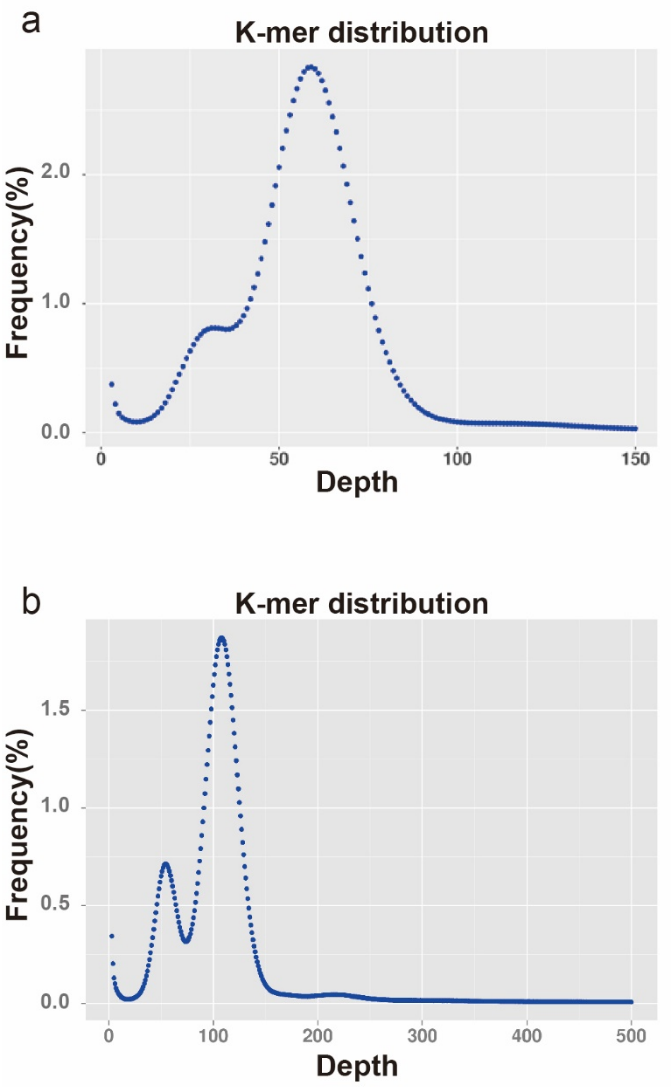

Supplementary Figure 1.

***K-mer* distribution of the *H. mohnikei* (a) and *H. zosterae* (b) genomes.** The *K-mer* spectrum was constructed based on a 19-*mer*. The figure shows the *K-mer* spectrum of raw reads. The x-axis represents the *K-mer* depth, whereas the y-axis represents the proportion of 19-mers with different coverage. The K_{num} of the *H. mohnikei* and the *H. zosterae* genomes were 38,103,526,114 and 57,501,085,335 based on the 19-mer. The K_{depth} were 58 and 107, respectively.

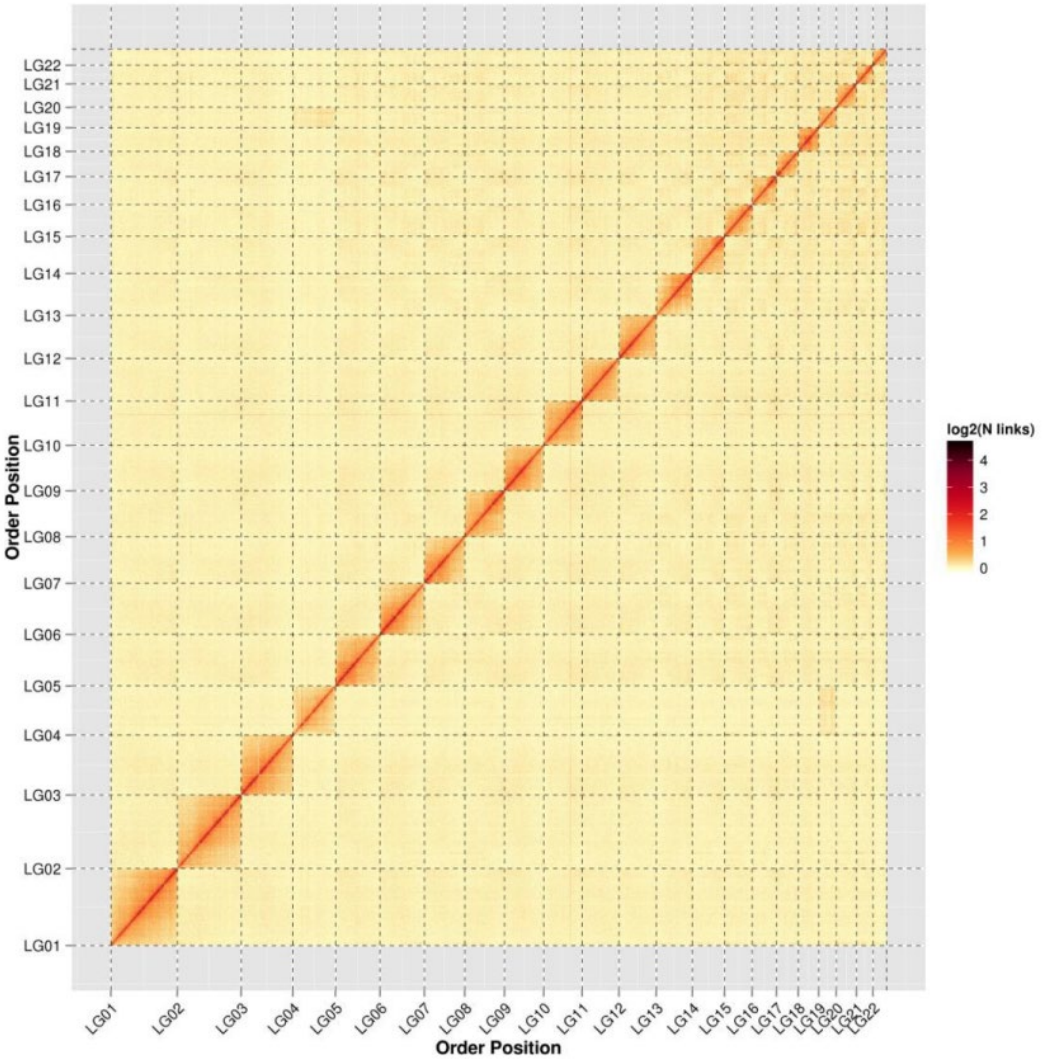

Supplementary Figure 2.

A Hi-C contact map of *H. zosterae* genome assembly. Chromosome-level genome assemblies comprising 22 chromosome-level scaffolds.

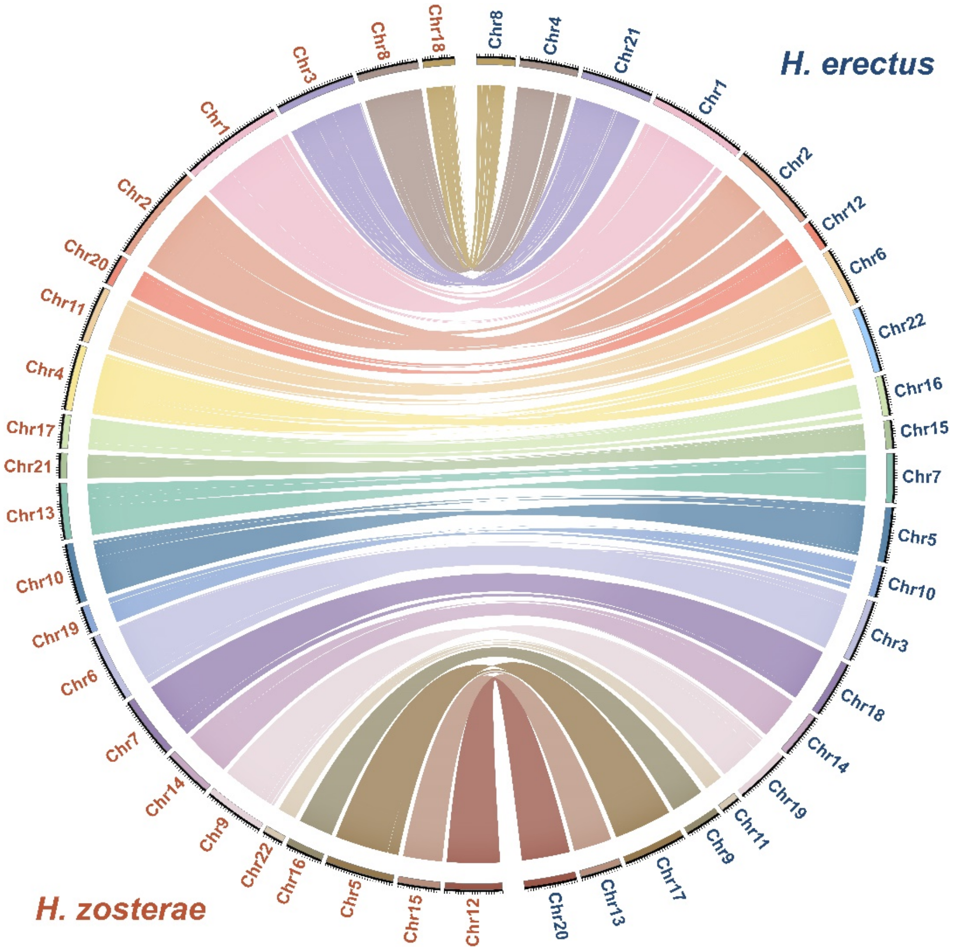

Supplementary Figure 3.

The chromosome synteny of *H. zosterae* v.s. *H. erectus*. Source data are provided as a Source Data file.

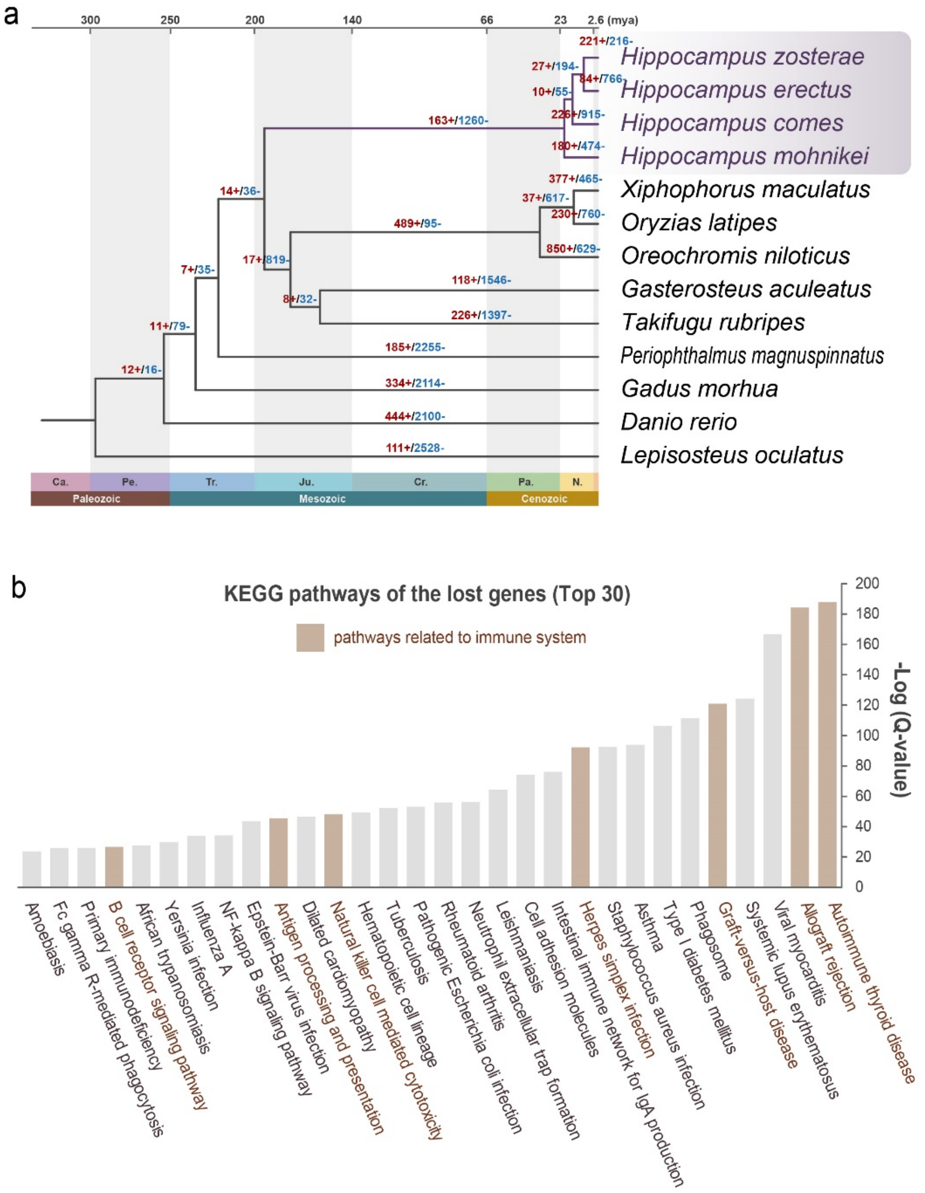

Supplementary Figure 4.

Contraction and loss of immune-related gene families in seahorses. (a) Expansion and contraction analyses of gene families of four seahorse species. The numbers near each branch indicate the number of significantly expanded (red) and contracted (blue) gene families. **(b)** Top 30 KEGG pathways of gene losses in seahorses (Q-value<0.01). Categories involved in immunity are colored in brown. The full list of categories is found in Supplementary Table 13, and Supplementary Data 3. Source data are provided as a Source Data file.

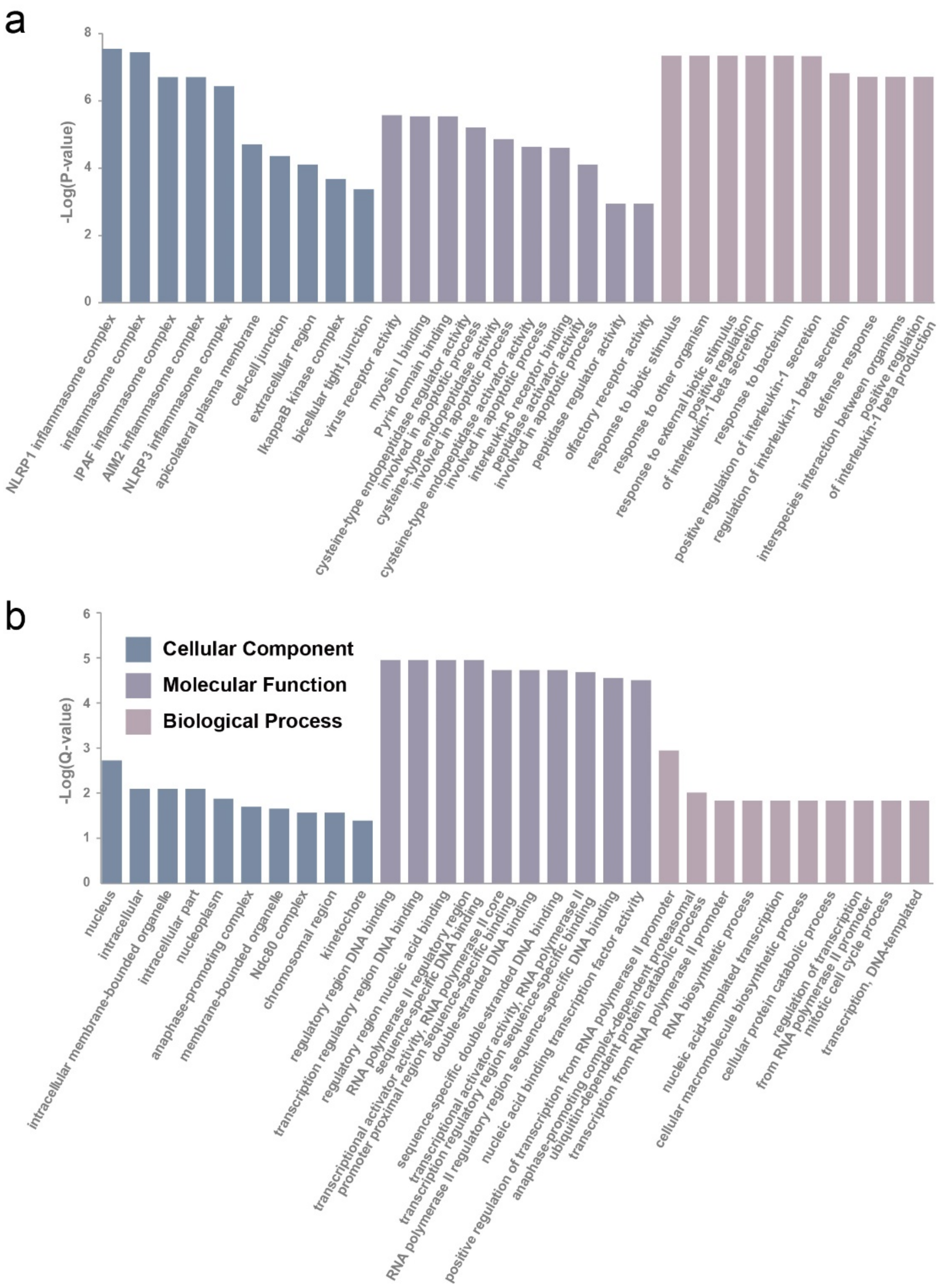

Supplementary Figure 5.

Gene Ontology (GO) analyses of the contracted gene families (a) and rapidly evolving genes (b) in the seahorse species. The top 10 significantly enriched GO terms are shown for cellular component (CC), molecular function (MF), and biological process (BP). The complete categories are listed in Supplementary Data 2 and Supplementary Data 6.

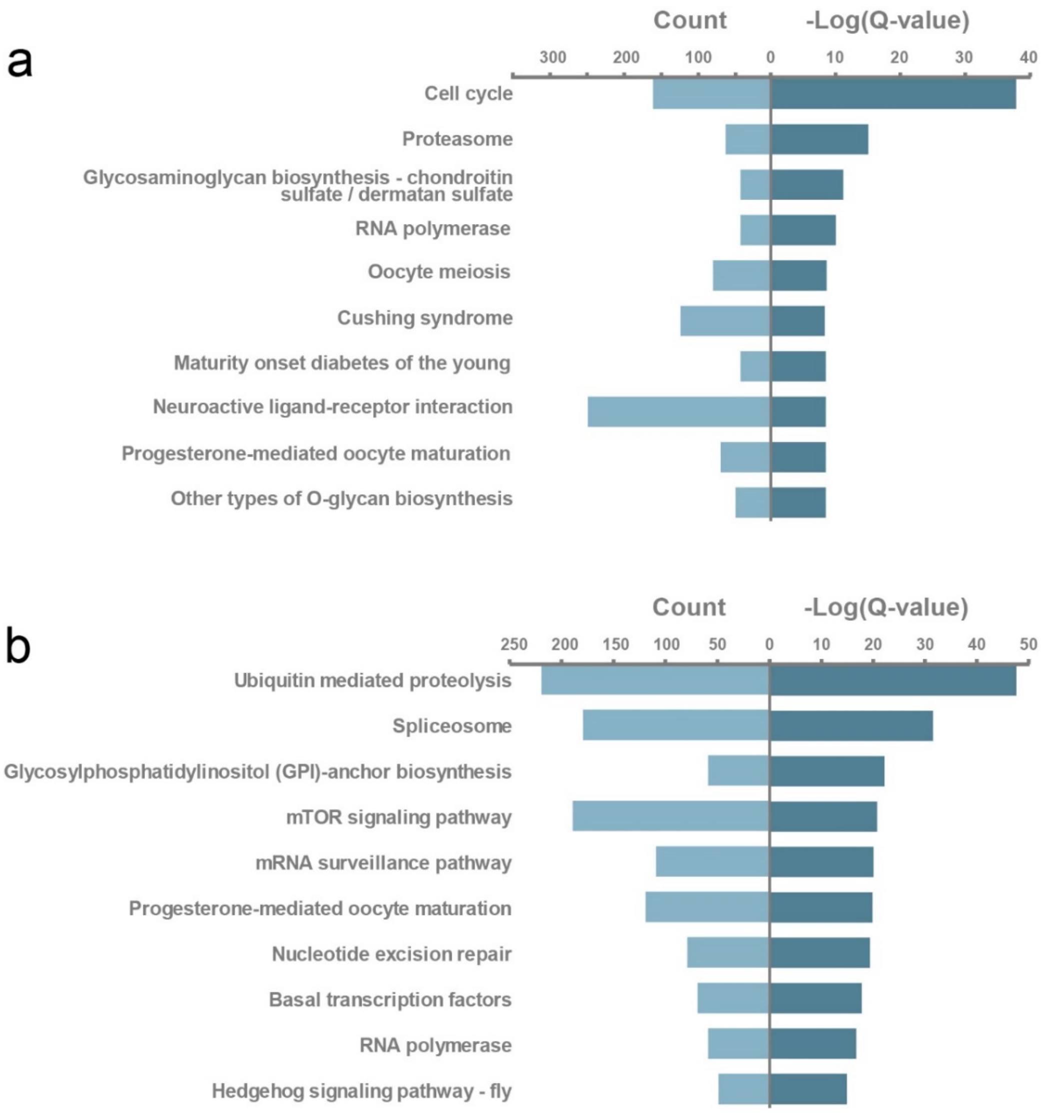

Supplementary Figure 6.

Top 10 enriched KEGG pathways of the rapidly evolving genes (a), and lineage-specific mutated genes (b) in the seahorse species. The horizontal axis (Count) indicates the number of genes enriched in each pathway. The Q-value statistical tests were two-sided and the FDR adjustments were made for multiple comparisons. The complete categories are listed in Supplementary Table 14-15.

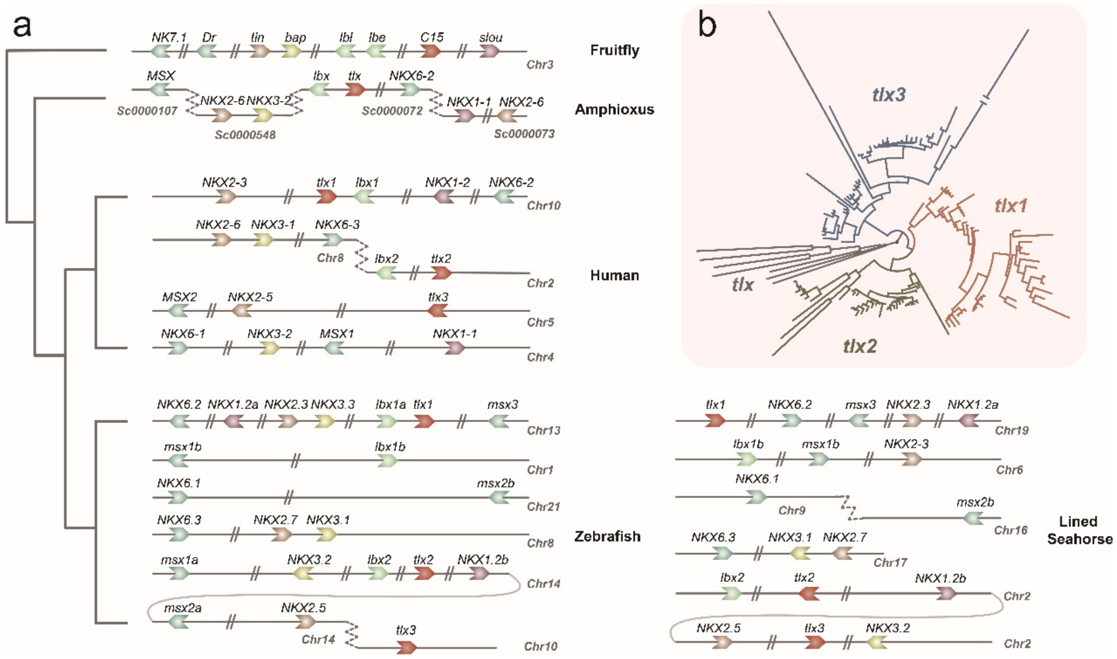

Supplementary Figure 7.

Local gene synteny (a) and phylogenetic analysis (b) of the tlx gene families. The results showed that the lined seahorses have intact *tlx* gene family members, including one copy of *tlx1*, *tlx2*, and *tlx3*, respectively. Source data are provided as a Source Data file.

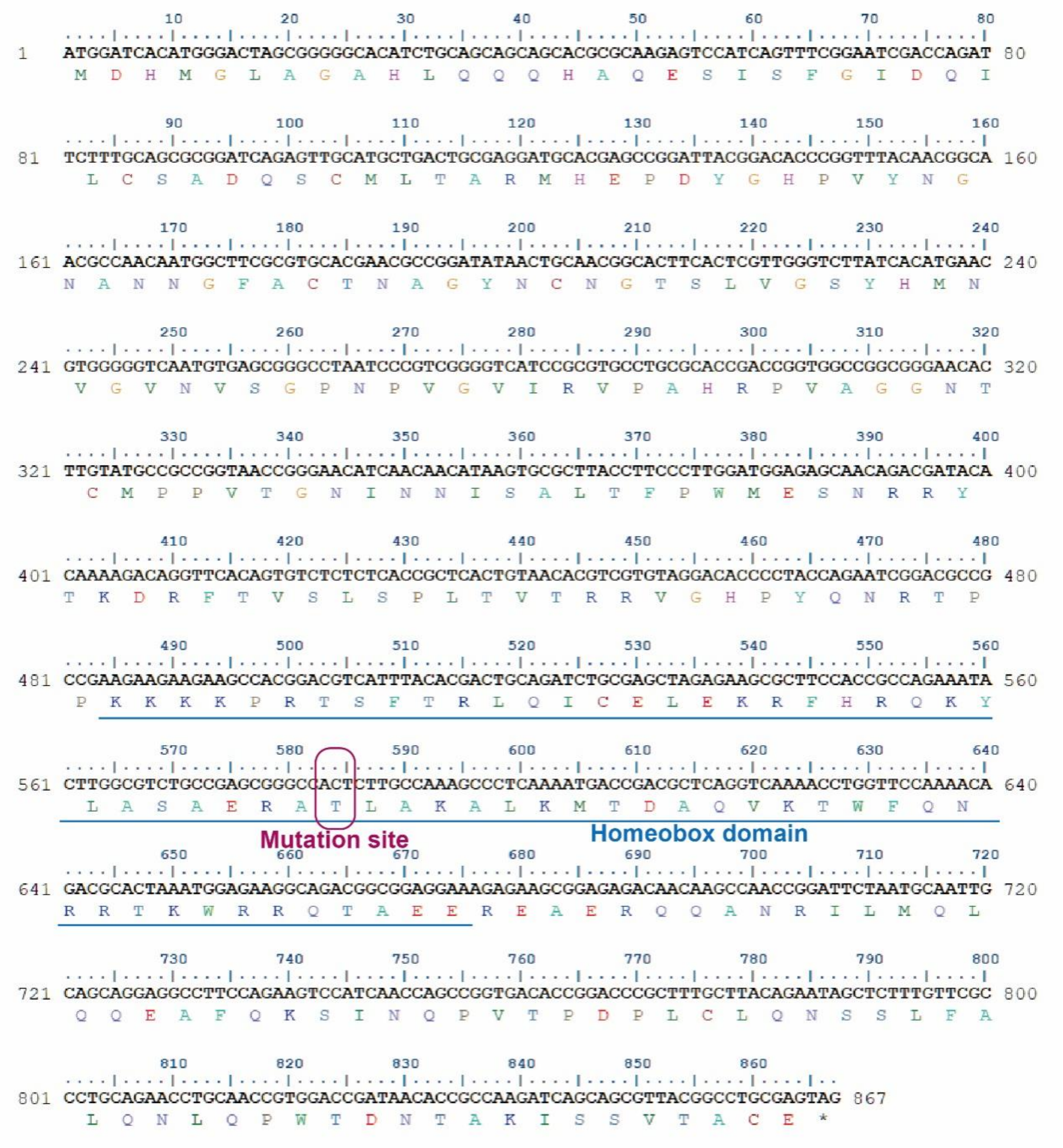

Supplementary Figure 8.

The ORF nucleotide sequences and the deduced amino acid sequences of *tlx1* in lined seahorse. The representative homeobox domain are underlined. The mutation site is indicated with a circle.

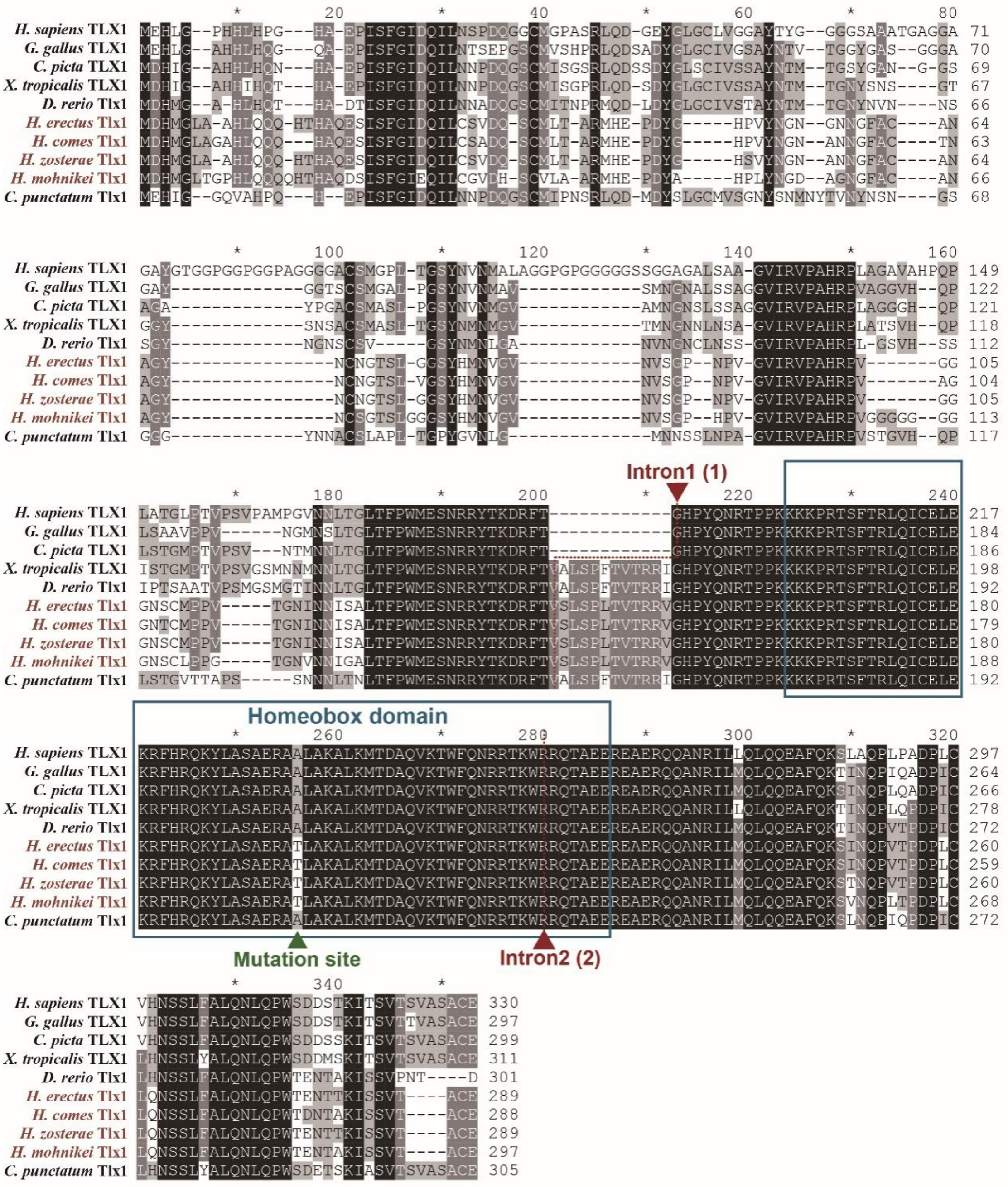

Supplementary Figure 9.

Alignment of the Tlx1 amino acid sequences of lined seahorse, tiger tail seahorse, dwarf seahorse, Japanese seahorse, and other representative vertebrates. The intron positions are indicated in the red triangle, and the homeobox domain is boxed in blue. The mutation sites in seahorses are marked with a green triangle.

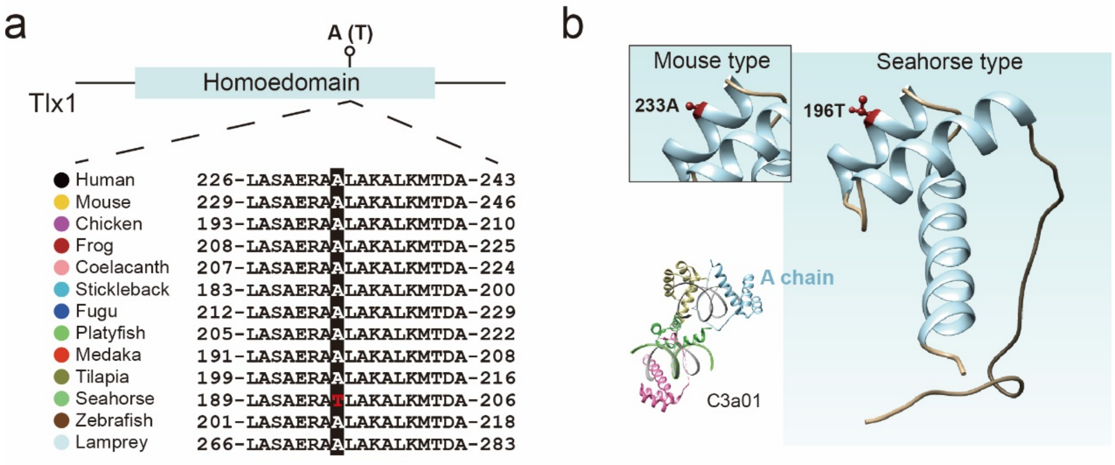

Supplementary Figure 10.

Specific mutation of *tlx1* in seahorse species. (a) Alignment of Tlx1 amino acids in the different representative vertebrate lineages**. (b)** 3D structure prediction of the lined seahorses.

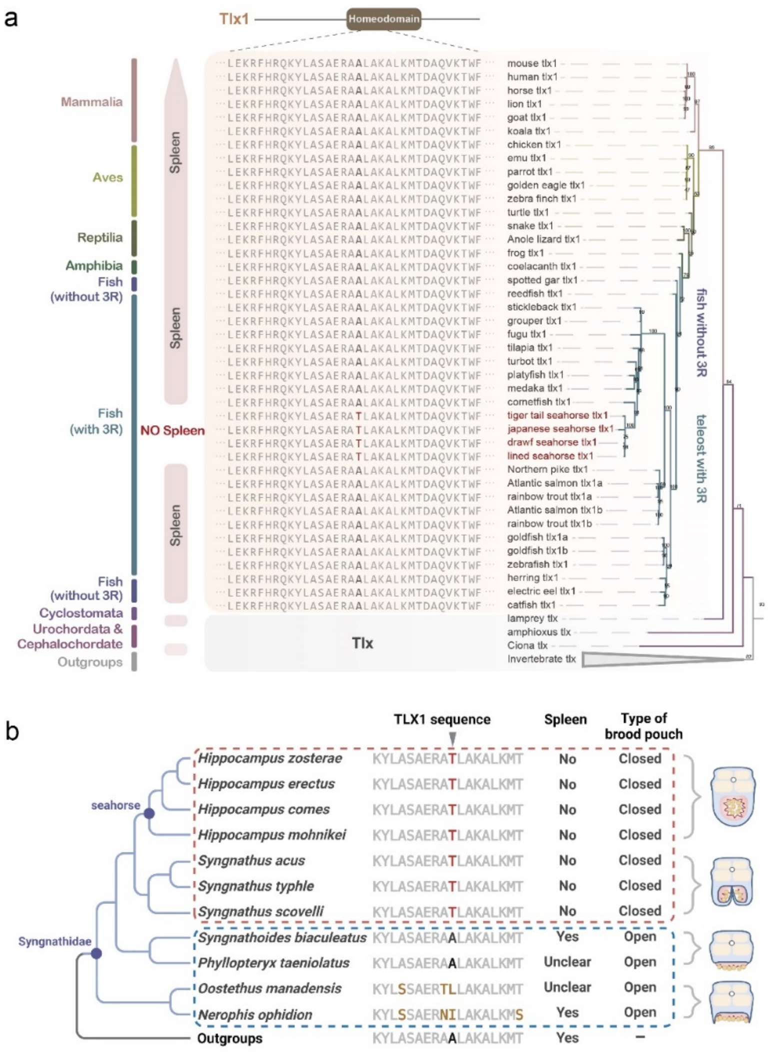

Supplementary Figure 11.

Multiple alignments of Tlx1 amino acids in different lineages. (a) Alignment of Tlx1 amino acids expanding a total of 38 vertebrates containing 23 fish species from 15 orders including four seahorse species (*Hippocampus*). **(b)** Multiple alignments of Tlx1 amino acids in Syngnathidae. *Hippocampus* and *Syngnathus* species have closed brood pouches and spleen loss, and exhibit A to T mutation in Tlx1. In contrast, seadragon (*Phyllopteryx taeniolatus*) and alligator pipefish (*Syngnathoides biaculeatus*) do not have this mutation, with the A preserved as in most other vertebrates. Meanwhile, *Oostethus manasensis* and *Nerophis ophidion* exhibited A to L and A to I, respectively. Source data are provided as a Source Data file.

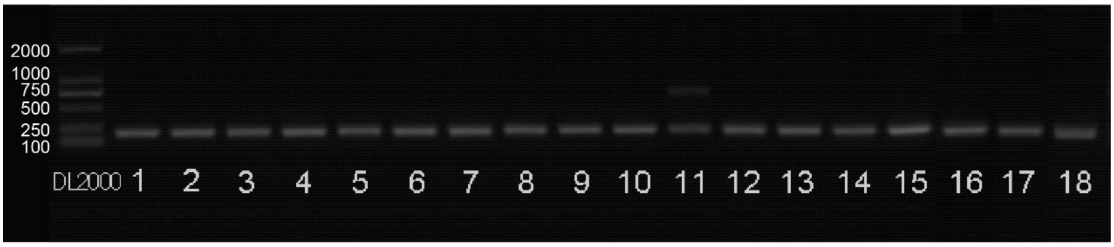

1, *H. abdominalis*
2, *H. kuda*
3, *H. capensis*
4, *H. algiricus*
5, *H. fuscus*
6, *H. reidi*
7, *H. jayakari*
8, *H. kelloggi*
9, *H. hippocampus*
10, *H. spinosissimus*
11, *H. camelopardalis*
12, *H. barbourin*
13, *H. mohnikei*
14, *H. zosterae*
15, *H. erectus*
16, *H. ingens*
17, *H. histrix*
18, *H. comes*

Supplementary Figure 12.

PCR validation of *tlx1* missense mutation in 18 seahorses. Degenerate primers were designed for *tlx1*, and *tlx1* can be amplified in all 18 seahorses. The experiment were conducted twice. Source data are provided as a Source Data file.

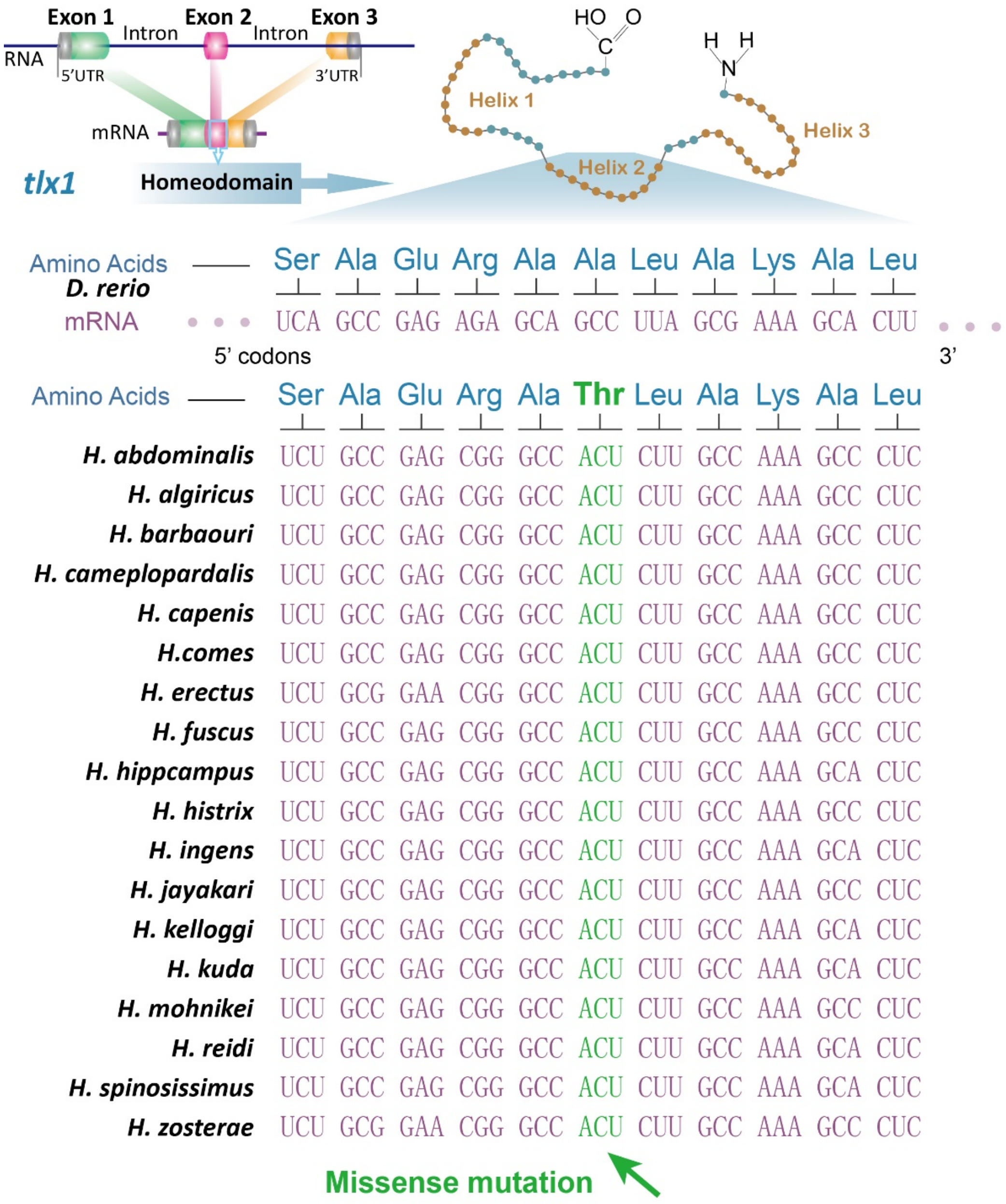

Supplementary Figure 13.

Gene structure analysis of *tlx1*, and PCR validation of the missense mutation in 18 seahorses. Further sequencing of all 18 seahorse species confirmed that seahorses share the identified mutation, as they all exhibit an Ala to Thr mutation. The missense mutation sites are highlighted in green.

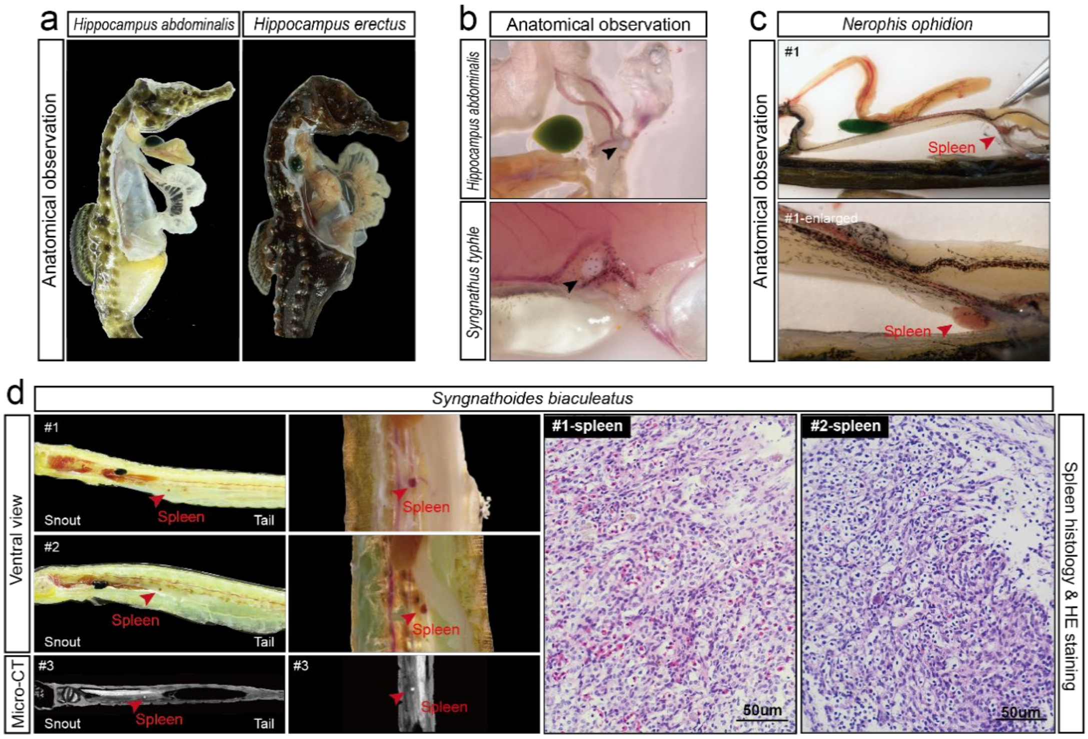

Supplementary Figure 14.

Splenic phenotypes detection of the (a-b) *Hippocampus* and *Sygnathus*, (c) *Nerophis ophidion* and (d) *Syngnathoides biaculeatus*. We provide splenic phenotypes of Syngnathides of anatomical photos, Micro CT scans and spleen histology. Specifically, *Syngnathoides biaculeatus* shows a spleen could unambiguously be identified (red arrowed), which is the same to *Nerophis ophidion*. However, we found a small white organ (black arrowed) in *Hippocampus* and *Sygnathus*, but the transcriptomic expression is quite different between it and the spleen, which implies it is not a functional spleen, and we termed it functional "asplenia". Due to the limitation of the sample, the histology analysis of the *Nerophis ophidion* was conducted one time, and the histology analysis of the *Syngnathoides biaculeatus* was conducted three times.

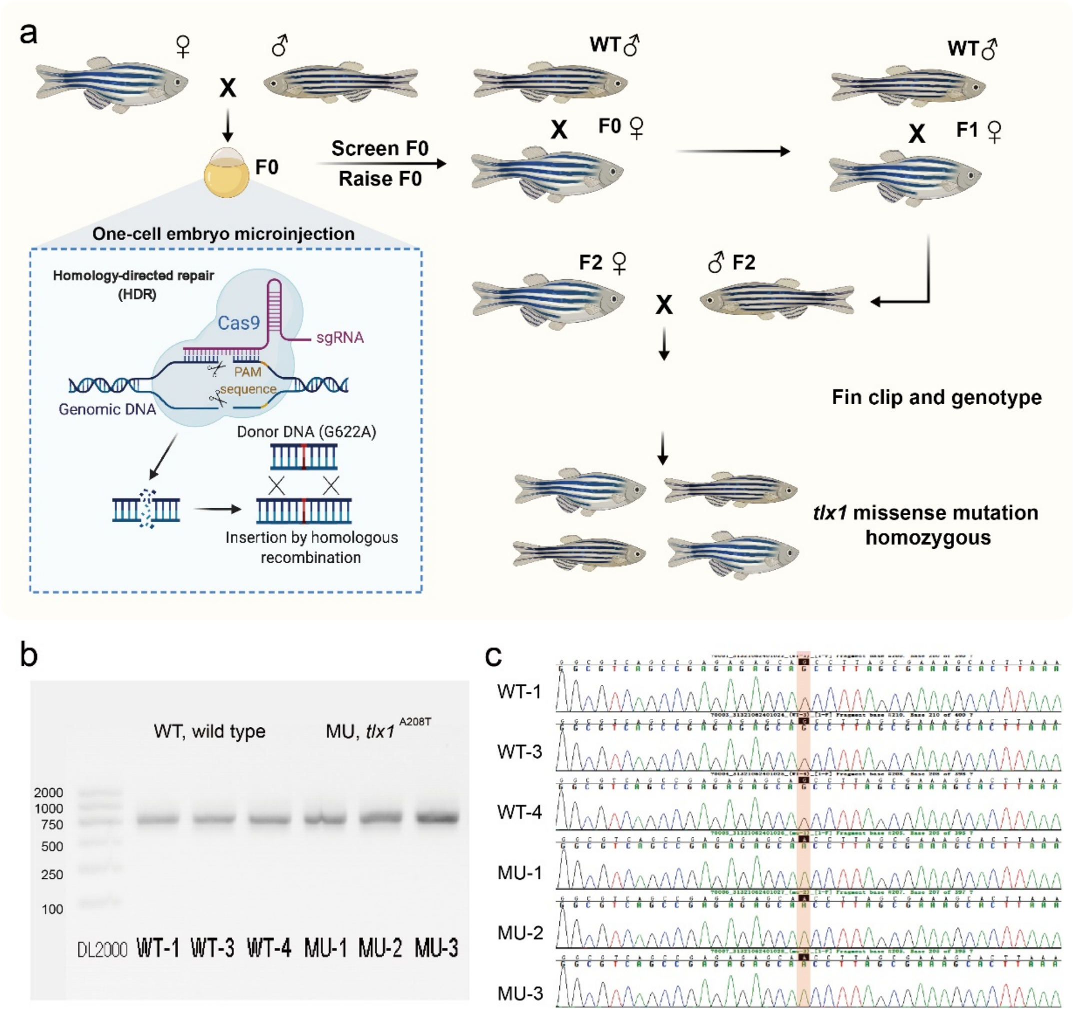

Supplementary Figure 15.

Establishment of *tlx1*-specific point-mutation (*tlx1*$^{\mathrm{A208T}}$) in zebrafish. (a) CRISPR/Cas9-mediated homologous recombination genome editing of *tlx1* G622A. ♀, female; ♂, male. **(b)** PCR validation of the *tlx1* point mutation in zebrafish. Degenerate primers were designed for *tlx1*, and *tlx1* was amplified in both WT and MU individuals (n=3). WT, wild type; MU, mutation. **(c)** Further sequencing of WT and MU *tlx1* also confirmed this mutation (site 622, G to A). Source data are provided as a Source Data file.

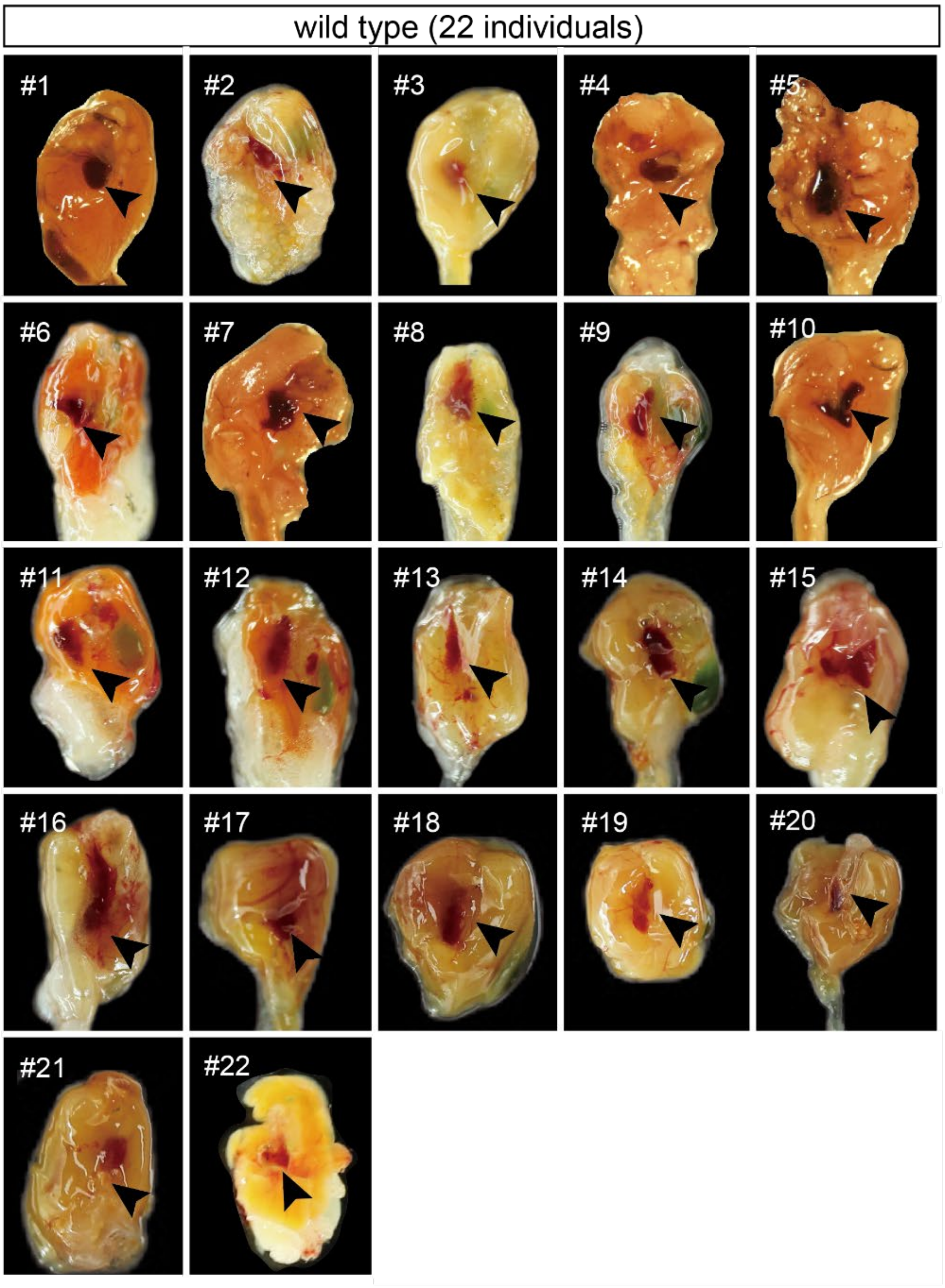

Supplementary Figure 16.

Photographs of the splenic phenotype in the wild-type zebrafishes (n=22). The spleens were indicated by the black arrow.

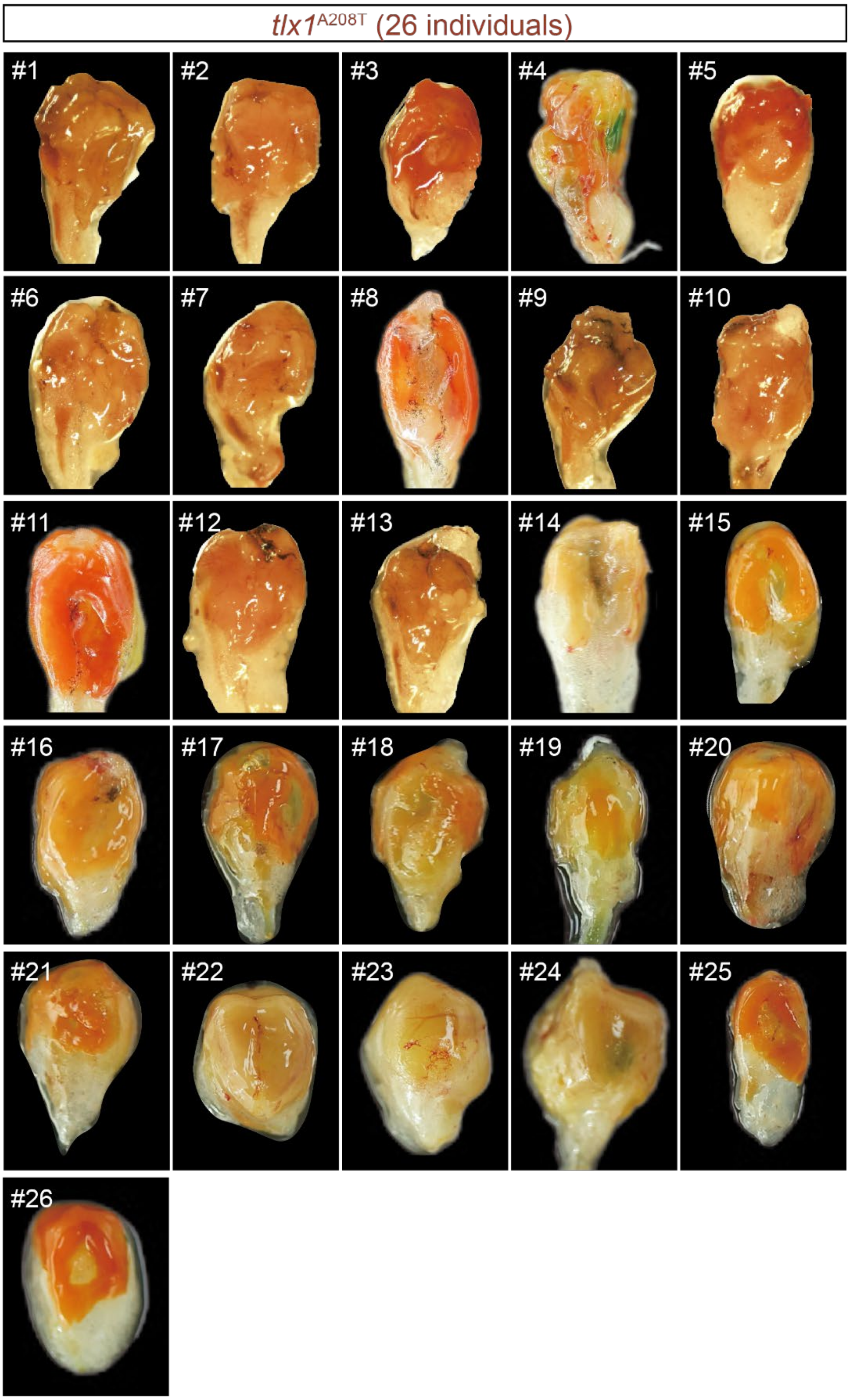

Supplementary Figure 17.

Photographs of the splenic phenotype in the *tlx1*^{A208T} type zebrafishes (n=26). All the detected *tlx1*^{A208T} zebrafishes exhibited asplenia.

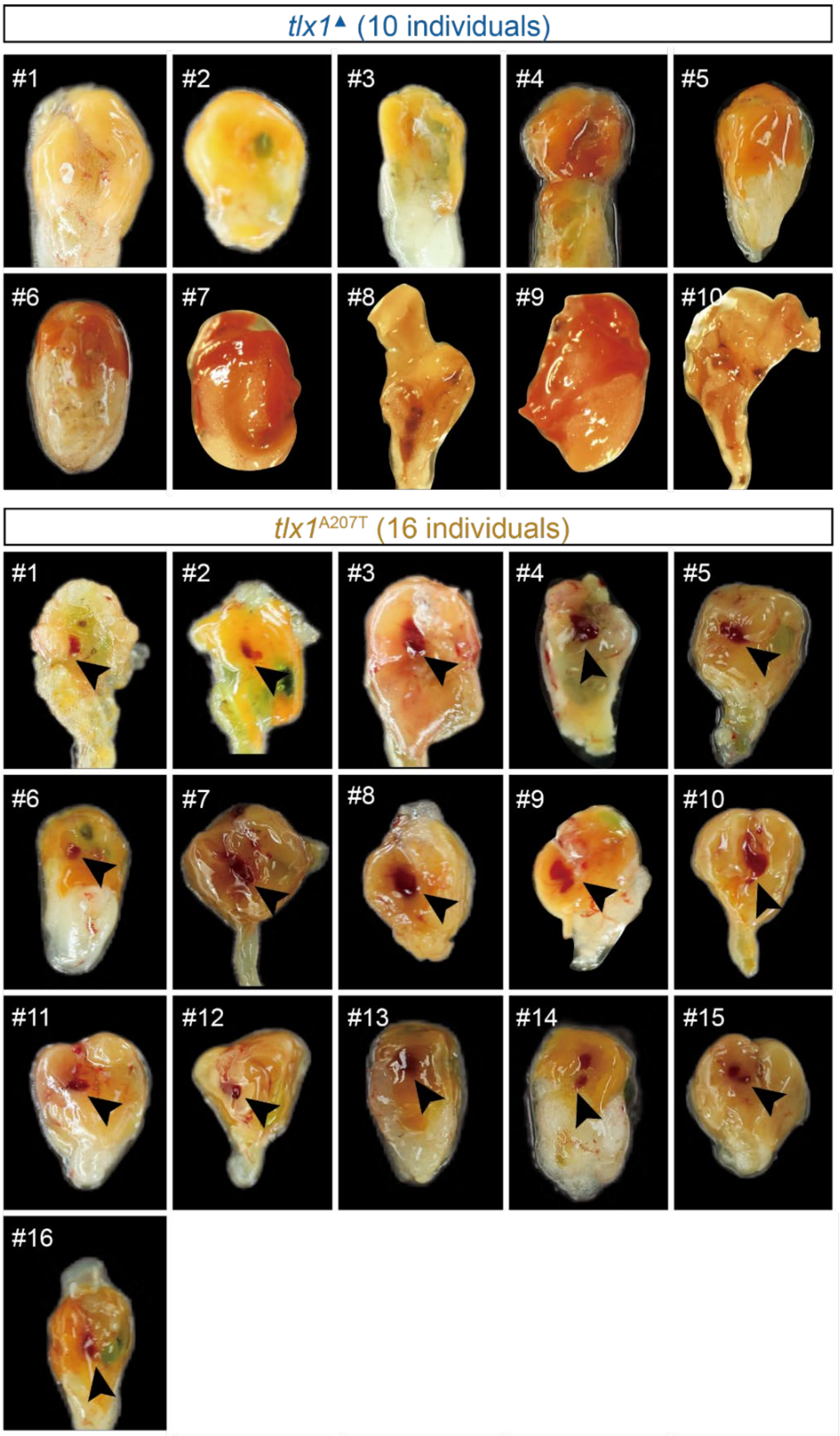

Supplementary Figure 18.

Photographs of the splenic phenotype in the *tlx1*▲ (n=10) and $tlx1^{A207T}$ (n=16) type zebrafishes. The spleens were indicated by the black arrow.

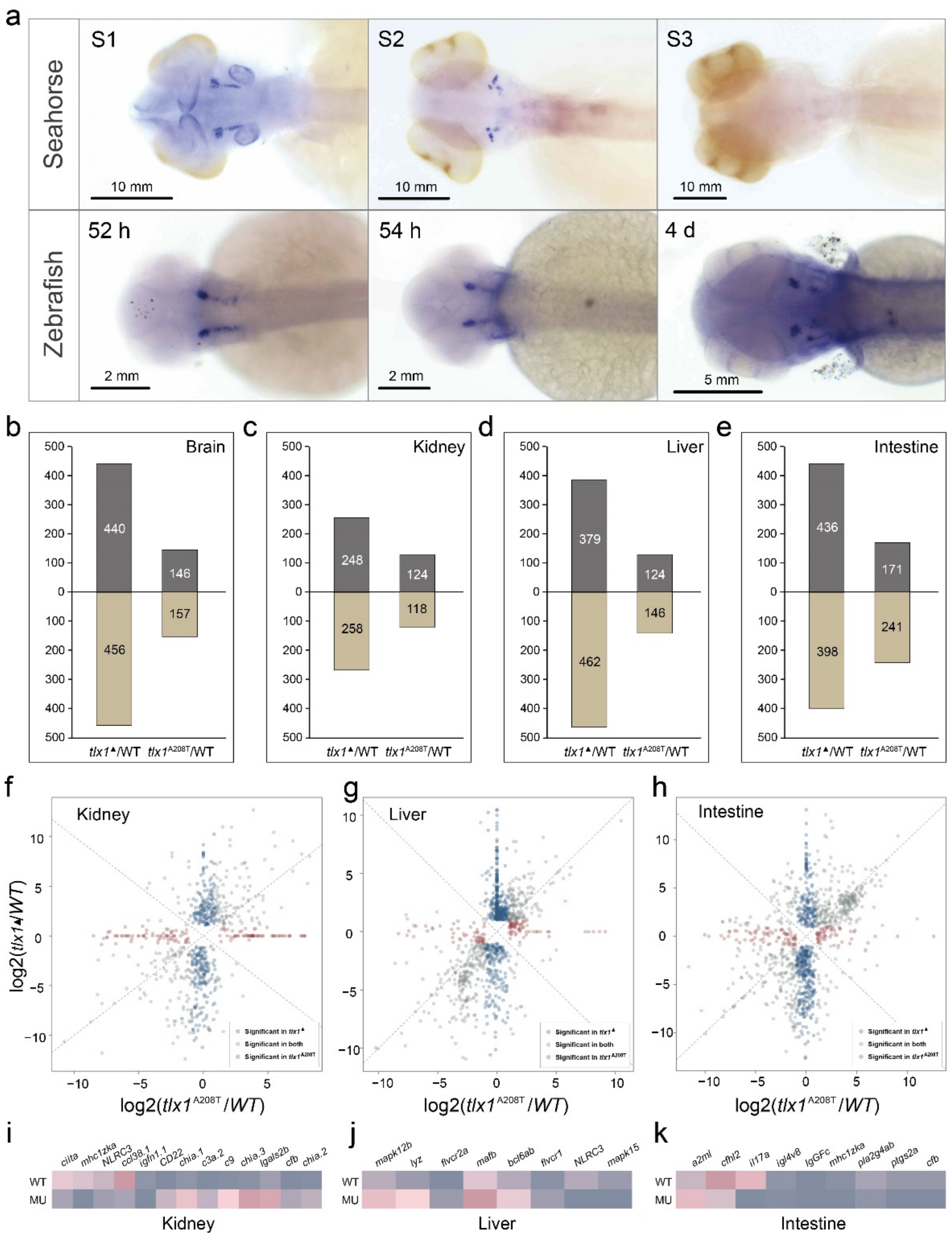

Supplementary Figure 19.

Whole mount *in situ* hybridization of seahorse *tlx1* and transcriptome analyses of wild-type, *tlx1*▲, and *tlx1*$^{\mathbf{A208T}}$ zebrafishes. (a) In seahorse embryos, *tlx1* was expressed in the hindbrain and pharyngeal arches at S1 and S2, but not at S3. Moreover, unlike zebrafish, no expression was detected in the corresponding splenic primordium region during embryogenesis. S1, early-stage, ~ 6 dpf; S2, mid-stage, ~12 dpf; S3, late-stage, ~ 18 dpf; h, hours; d, days. All these *in situ* hybridization analyses

were conducted twice. **(b-e)** Histogram of DEGs in the brain, kidney, liver, and intestine in *tlx1*▲ vs. WT and *tlx1*^{A208T} vs. WT groups, respectively. The *tlx1*^{A208T} line always had transcriptomic patterns more similar to those of the wild type. Gray color, upregulated genes; brown color, downregulated genes. **(f-h)** Plot analysis showing the mRNA levels of DEGs between *tlx1*▲ and *tlx1*^{A208T}. Gene selection criteria for b-h are described in the Methods. **(i-k)** Heatmap of immune-related genes in kidney, liver, and intestine between wild type and *tlx1*^{A208T} zebrafishes.

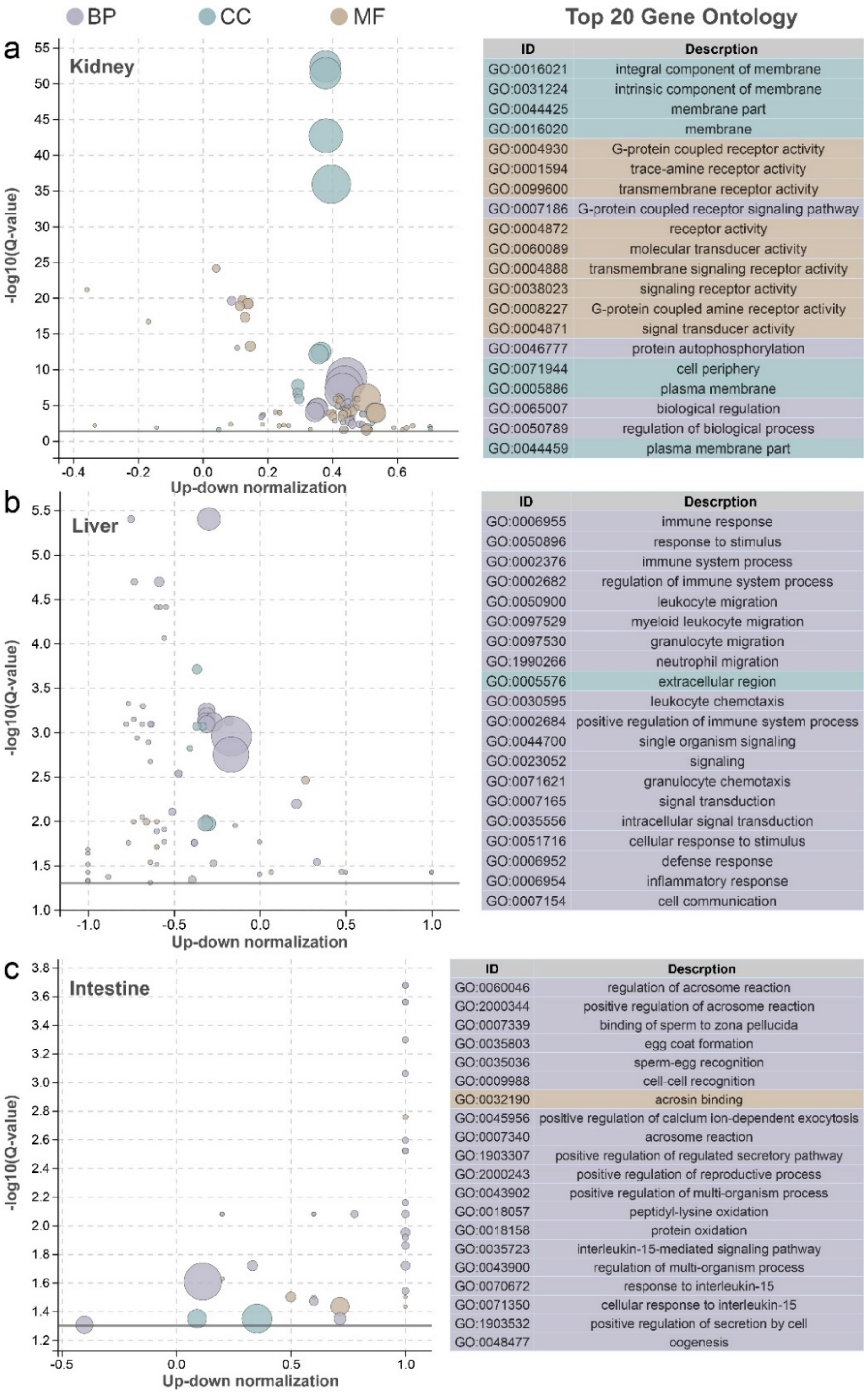

Supplementary Figure 20.

Top 20 most-enriched Gene Ontology pathways in the kidney (a), liver (b), and intestine (c) based on Q-value. The size of the circle represents the number of genes in each Gene Ontology term. CC, cellular component; BP, biological process; MF, molecular function.

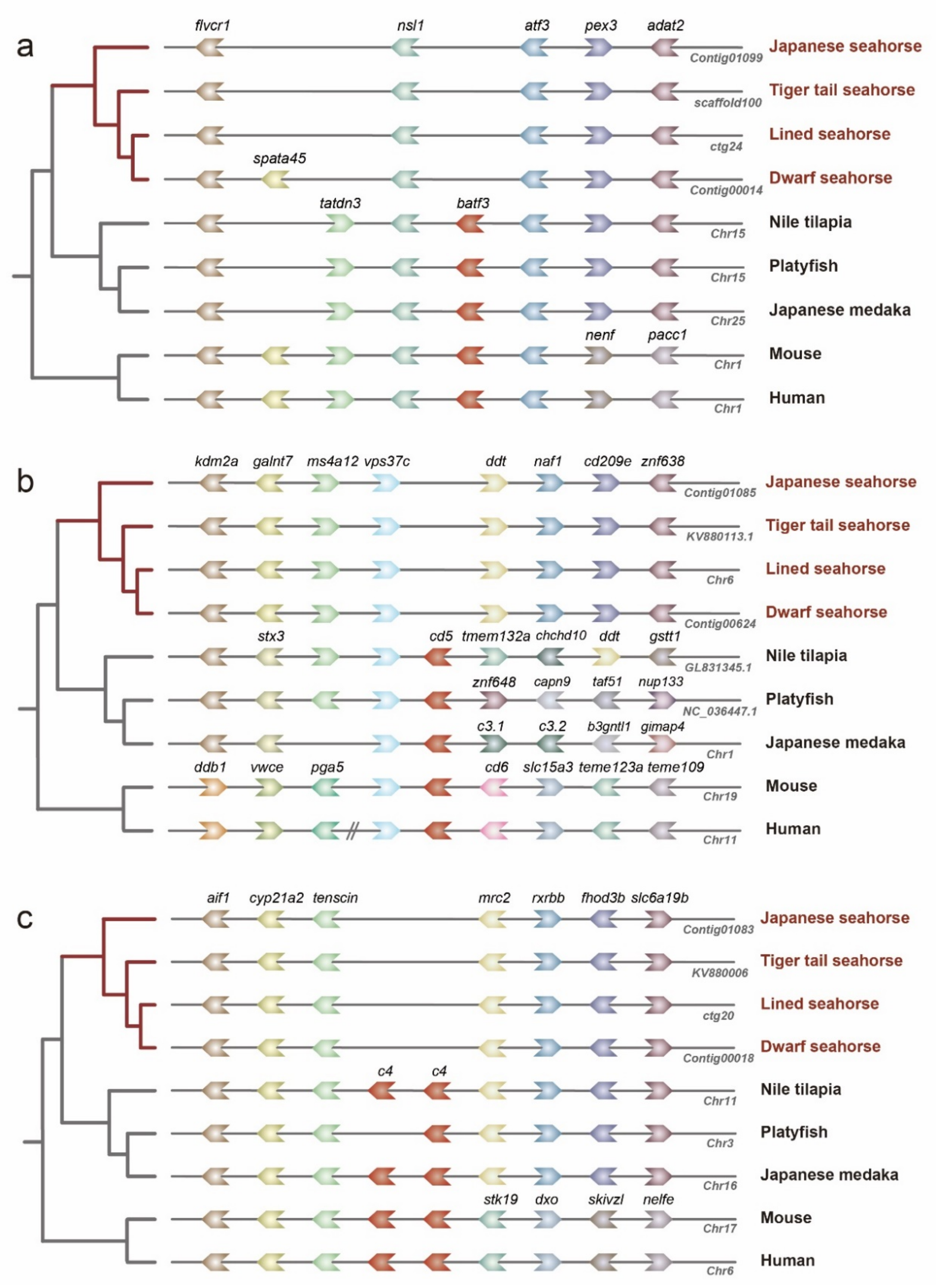

Supplementary Figure 21.

Syntenic analysis of the *batf3* (a), *CD5* (b), and *C4* (c) genes in four seahorses (Japanese seahorse, tiger tail seahorse, lined seahorse, and dwarf seahorse) and other representative fishes. Source data are provided as a Source Data file.

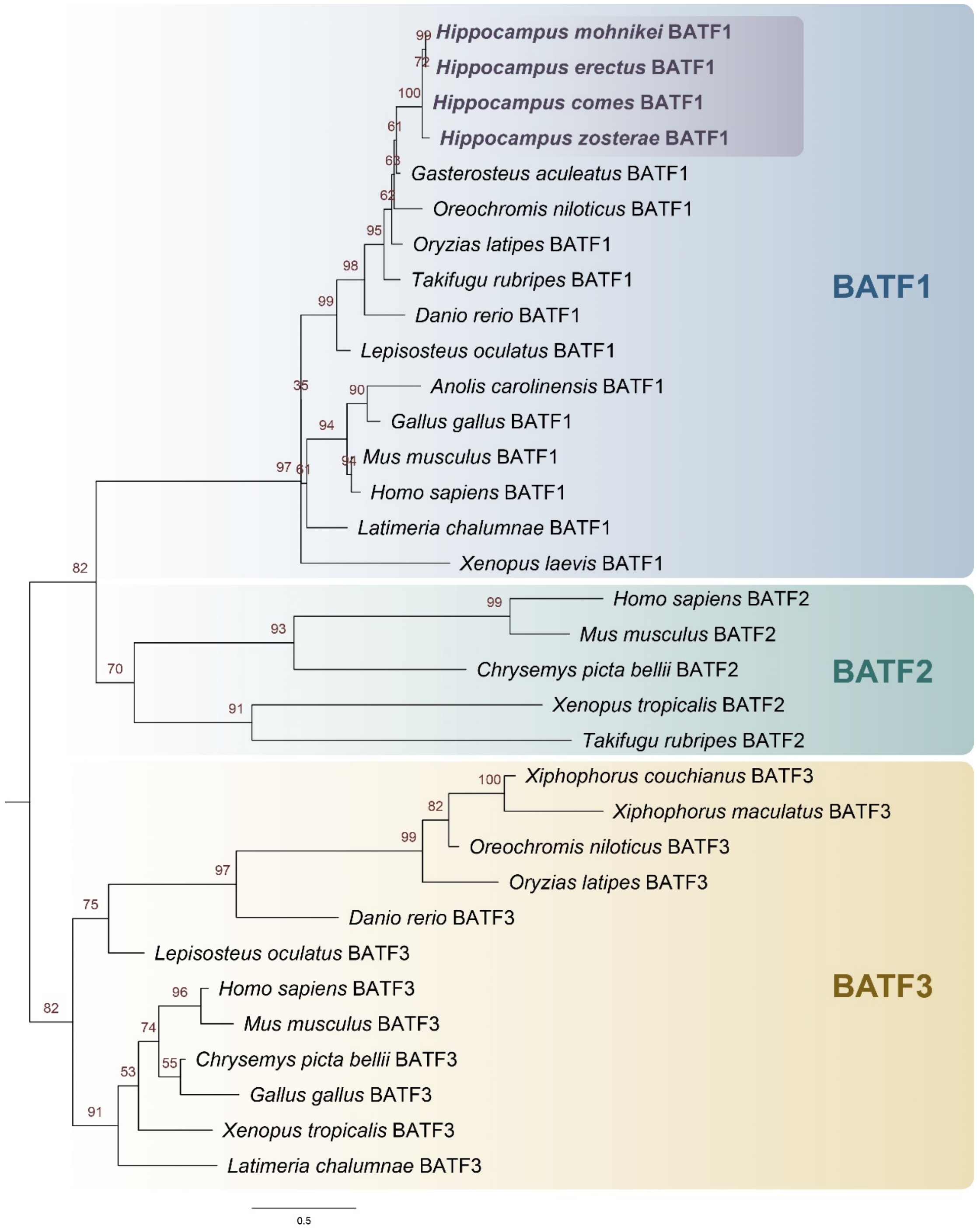

Supplementary Figure 22.

Phylogenetic tree of the *batf* gene family in vertebrates shows the absence of *batf3* in seahorses. *Hippocampus* species are highlighted in purple shade. Source data are provided as a Source Data file.

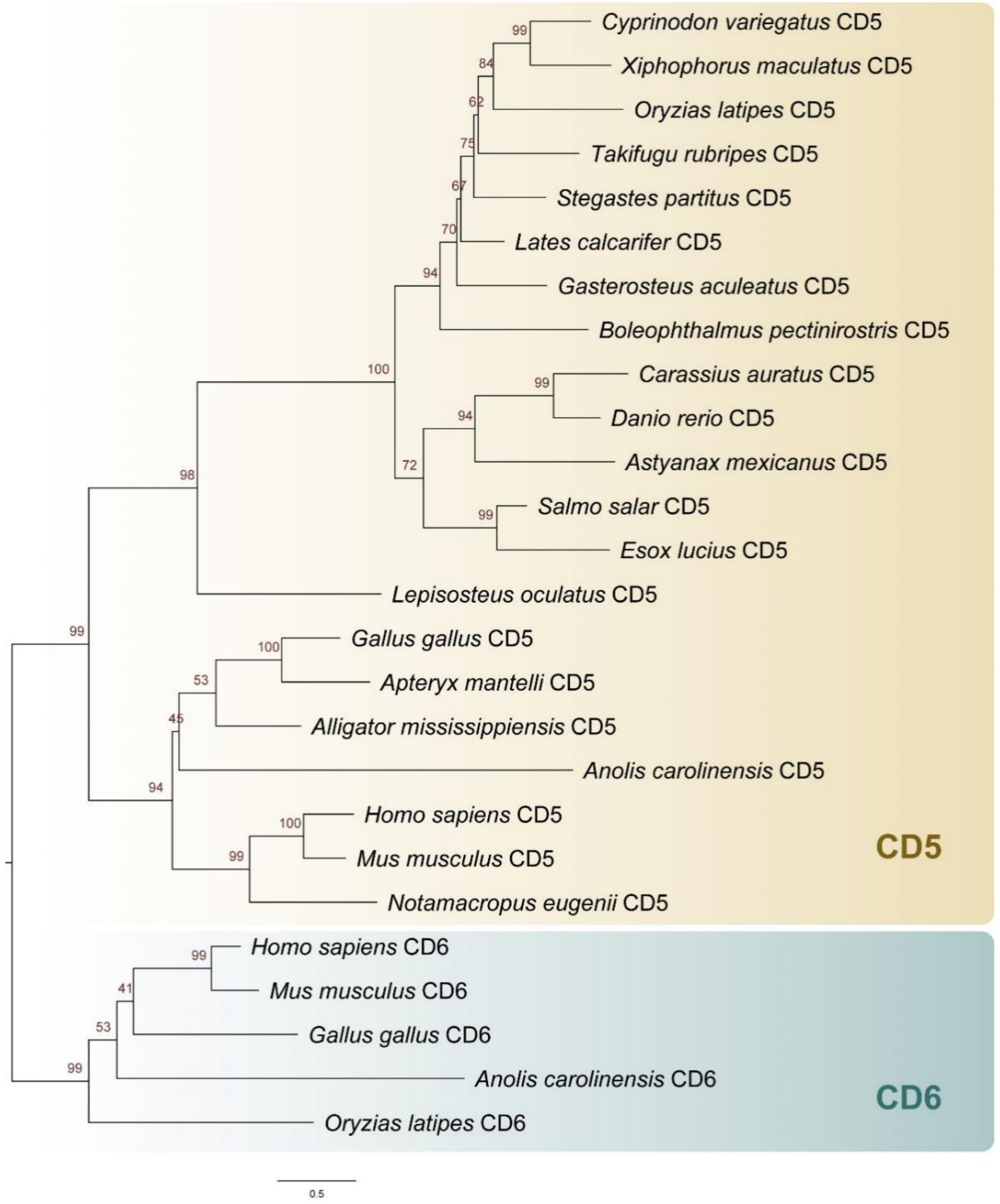

Supplementary Figure 23.

Phylogenetic tree of the *CD5* and *CD6* gene families in vertebrates shows the absence of *CD5* in seahorses. Source data are provided as a Source Data file.

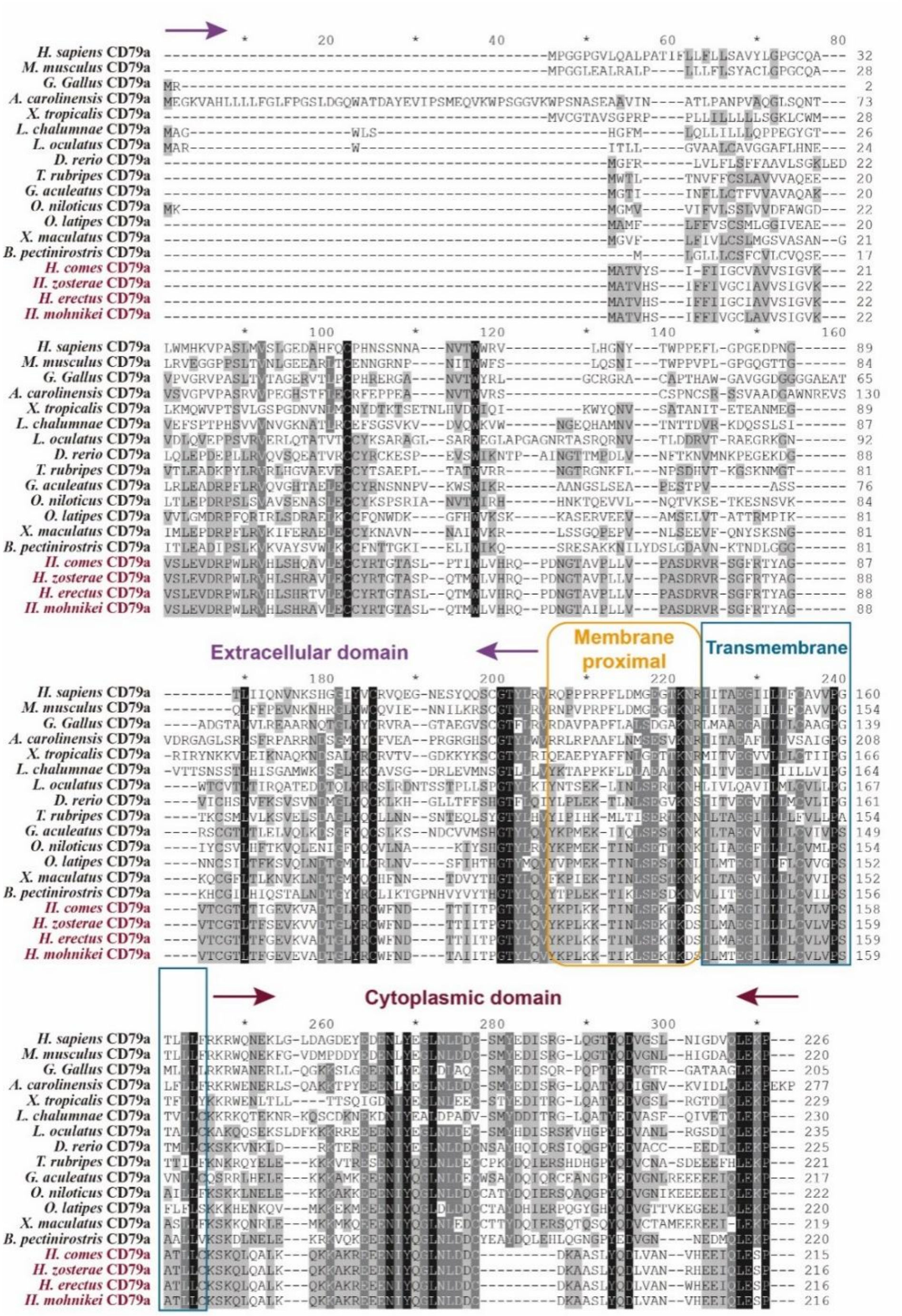

Supplementary Figure 24.

Alignment of the CD79a amino acid sequences of lined seahorse, tiger tail seahorse, dwarf seahorse, Japanese seahorse, and other representative vertebrates. The structure of CD79a is shown, including the extracellular domain (purple), membrane proximal part (yellow), transmembrane region (blue), and cytoplasmic domain (red).

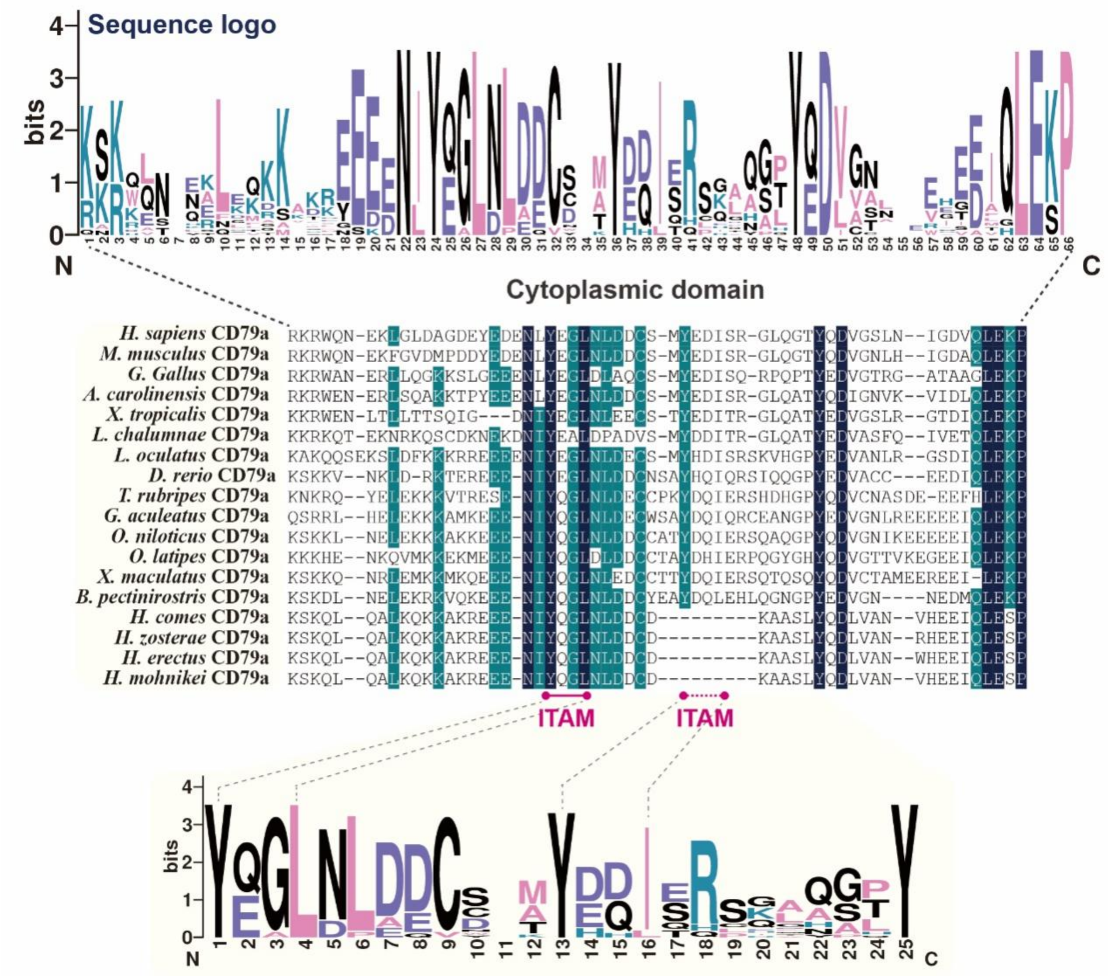

Supplementary Figure 25.

Sequence logo and amino acid alignment of the CD79a cytoplasmic domain of lined seahorse, tiger tail seahorse, dwarf seahorse, Japanese seahorse, and other representative vertebrates. The CD79a cytoplasmic domain contains two conserved dual phosphotyrosine binding motif, termed immunotyrosine-based activation motif (ITAM). One necessary ITAM motif in CD79a is missing in the four seahorse species. N, NH2-terminus; C, COOH-terminus. Source data are provided as a Source Data file.

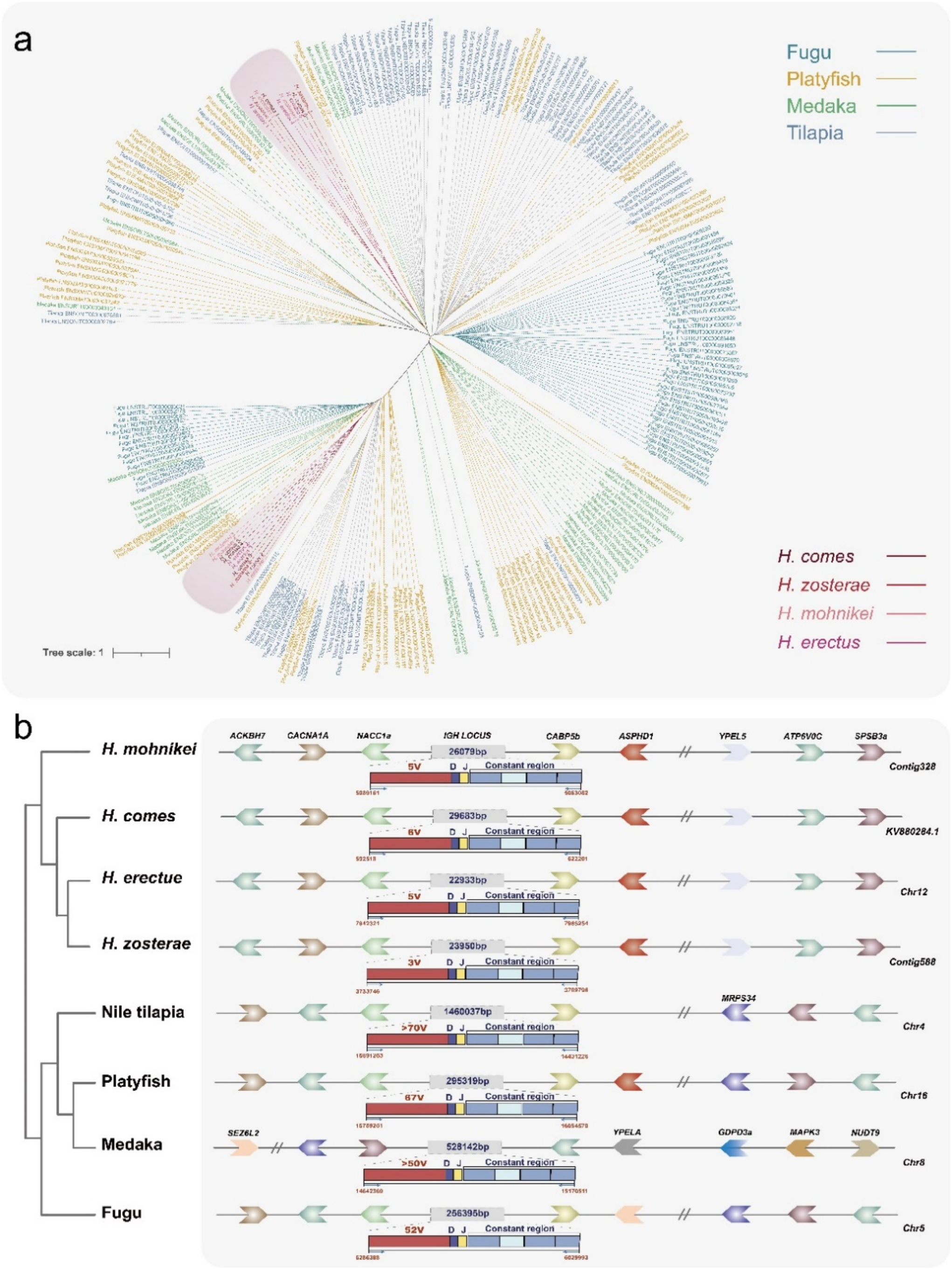

Supplementary Figure 26.

Phylogenetic and gene microsynteny analyses of the *ighv* genes in Japanese seahorse, tiger tail seahorse, lined seahorse, dwarf seahorse, and other representative fishes. (a) Seahorses have significantly reduced number of *ighvs*. *Hippocampus* species are highlighted in red shade. **(b)** In four seahorses scaffold length of *ighvs* is significantly reduced (ranging from 22,933 to 29,683 bp) compared

to that in other representative fishes (ranging from 256,395 to 1,460,037 bp). V, variable, D, diversity, J, joining. Source data are provided as a Source Data file.

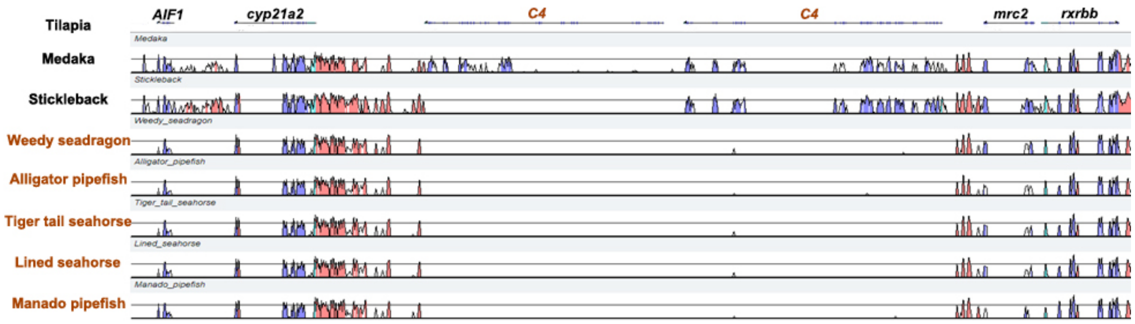

Supplementary Figure 27.

Vista plot of conserved elements in the *C4* syntenic region in tilapia (reference genome), medaka, stickleback, weedy seadragon, alligator pipefish, tiger tail seahorse, lined seahorse, and Manado pipefish. The *C4* gene is missing in the Syngnathidae family.

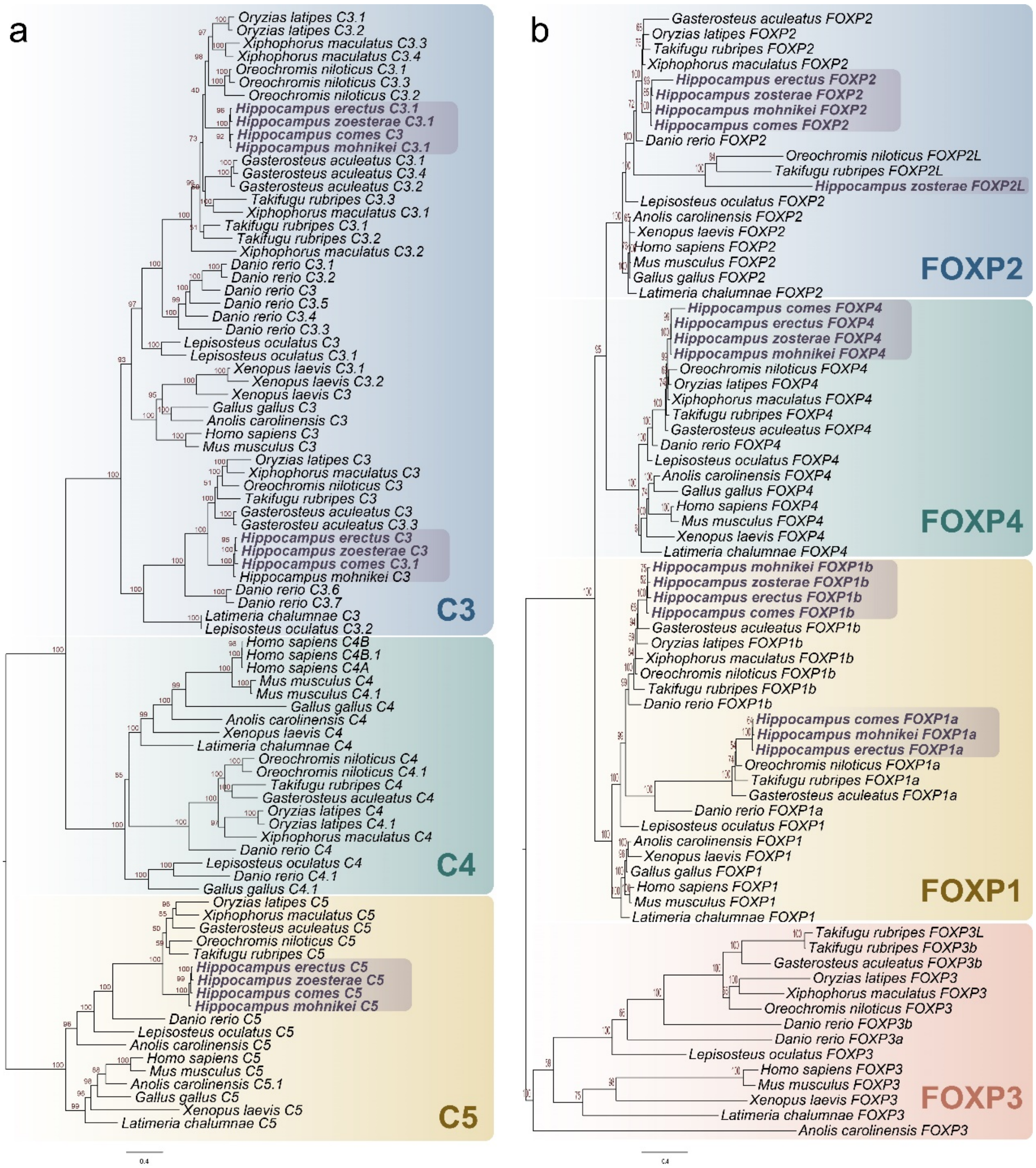

Supplementary Figure 28.

Phylogenetic tree analyses of the *C3*, *C4,* and *C5* gene families (a), as well as the *foxp* gene family (b) in vertebrates. *Hippocampus* species are highlighted in purple shade. Source data are provided as a Source Data file.

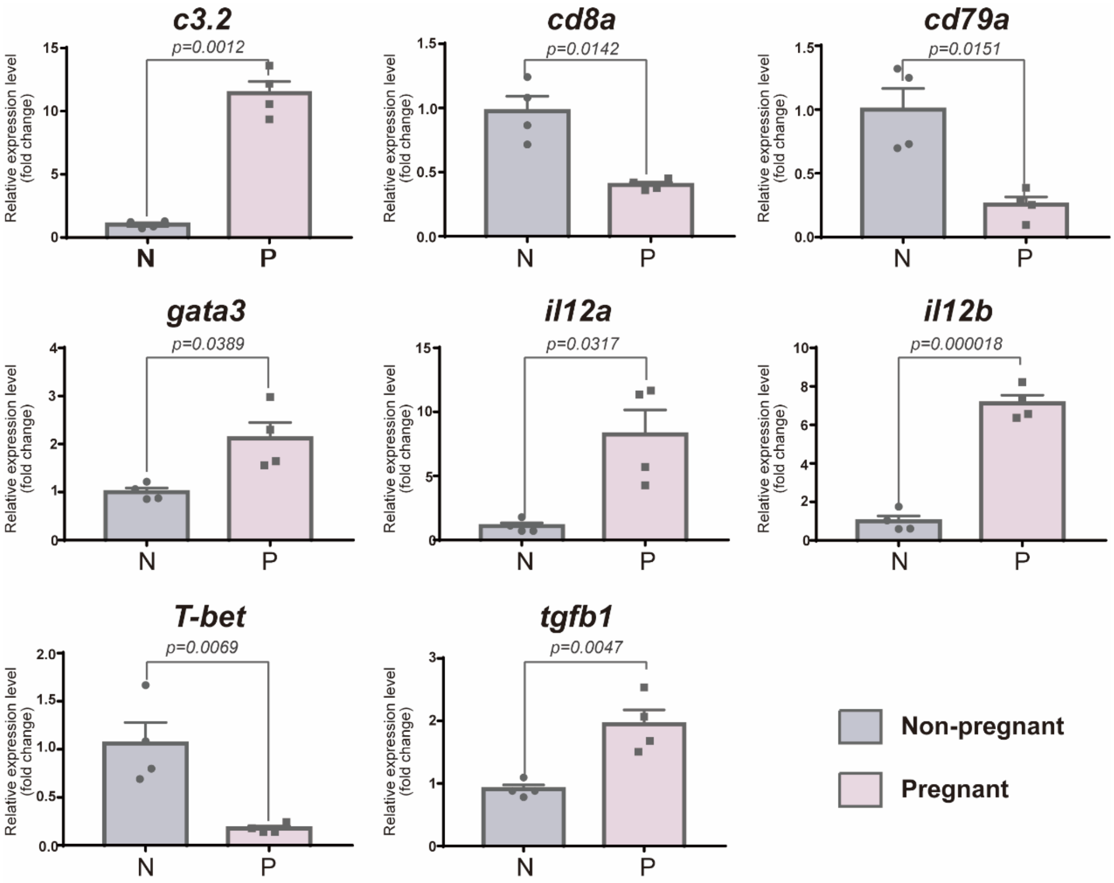

Supplementary Figure 29.

Temporal-spatial expression analysis of the genes involved in pregnancy in male lined seahorse by qRT-PCR. Adult male non-pregnant and pregnant lined seahorses (each group including n=4 biologically independent samples) were used to derive statistics. Significant differences are represented by *P* value using t-test. *β-actin* is used as an internal control, and the data are presented as the mean ± SEM. N, non-pregnant; P, pregnant.

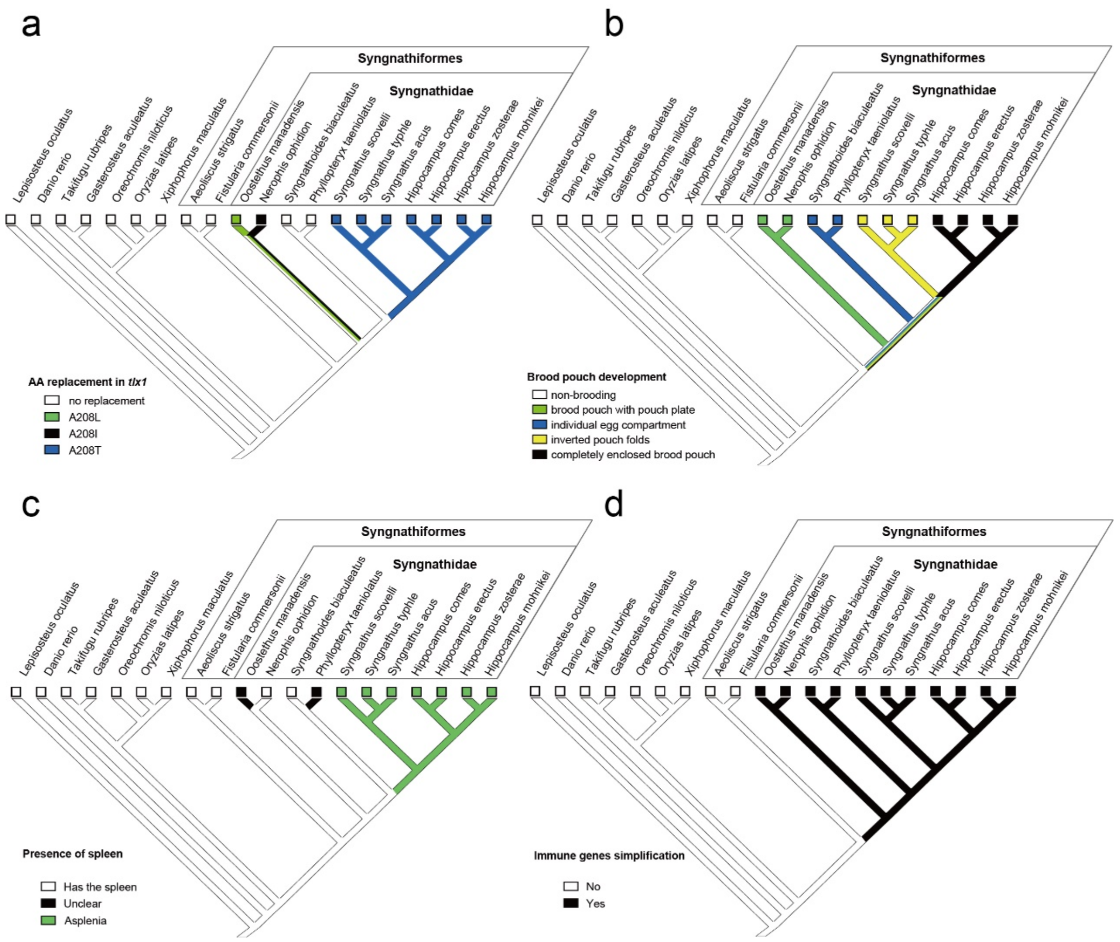

Supplementary Figure 30.

Most parsimonious reconstruction of (a) AA replacement in *tlx1*, (b) brood pouch development, (c) presence of spleen and (d) immune genes simplification, mapped on the consensus phylogenetic tree using Mesquite Ver.3.70 (http://www.mesquiteproject.org/). The empirical data coded as boxes where available. Character states of the splenic phenotype for *Syngnathus acus* and *Syngnathus scovelli* derived from the same genera *Syngnathus typhle*.

Supplementary Table 1. ***Hippocampus spp.*** **samples used for genome and Hi-C sequencing.**

Species	Sampling origin	Sampling date	Sex	Genome sequencing	Hi-C sequencing	Note
Hippocampus mohnikei	Rizhao, Shandong, China	2017	male	muscle	no	newly sequenced
Hippocampus zosterae	Florida, United States	2015	male	muscle	blood	newly sequenced
Hippocampus erectus	Zhanjiang, Guangdong, China	2016	male	muscle	muscle	referenced Li et al., 2021[24]
Hippocampus comes	Singapore	2014	male	muscle	no	referenced Lin et al., 2016[25]

Supplementary Table 2. Genome sequencing data by Illumina.

**Sample ID**	**Library**	**Data (Gb)**	**Depth (×)**
H. mohnikei	350 bp	44	74.84
H. zosterae	350 bp	65	137.62

Supplementary Table 3. Genome size estimation with *K-mer* distribution analysis based on a 19-mer.

Sample ID	Genome size (Mb)	Heterozygosity (%)	Repeat ratio (%)
H. mohnikei	580.80	0.58	40.72
H. zosterae	474.90	0.92	27.46

Supplementary Table 4. Raw reads data generated for *H. mohnikei* and *H. zosterae* genomes.

Species	Platform	Reads Num	Total bases (bp)	N50 (bp)	Mean (bp)
H. mohnikei	MinION	2,125,134	54,188,920,124	34,657	25,499
H. zosterae	PacBio Sequel	5,278,497	49,994,997,251	14,749	9,471

Supplementary Table 5. Summary of genome sizes and assembly statistics.

Species	Genome size	Sequencing coverage	Assembly size (bp)	N50 contig (bp)	Complete BUSCOs (%)	Fragmented BUSCOs (%)	Missing BUSCOs (%)
H. mohnikei	580	103 ×	527,386,034	18,090,544	93.46	2.07	4.47
H. zosterae	458	109 ×	458,253,157	7,285,248	92.95	2.88	4.97

Supplementary Table 6. Statistics of the anchored chromosomes for *H. zosterae* genome.

Chromosome ID	Contig Number	Length (bp)
Chr01	13	37,900,890
Chr02	22	36,583,144
Chr03	11	29,635,505
Chr04	45	27,599,681
Chr05	7	25,462,395
Chr06	10	25,305,228
Chr07	15	23,110,276
Chr08	8	22,732,700
Chr09	10	22,516,970
Chr10	13	22,092,442
Chr11	9	20,979,183
Chr12	8	21,063,346
Chr13	16	20,879,188
Chr14	4	18,106,957
Chr15	12	15,981,988
Chr16	3	13,895,953
Chr17	5	12,438,740
Chr18	17	12,223,714
Chr19	14	11,528,794
Chr20	6	11,550,811
Chr21	5	9,437,239
Chr22	11	8,288,061
Anchored length (%)		449,313,205 (98.05%)

Supplementary Table 7. Statistics of TEs in *H. mohnikei* genome.

Type	Number	Length (bp)	Rate (%)
ClassI/DIRS	5,674	2,388,801	0.45
ClassI/LARD	325,491	88,036,891	16.69
ClassI/LINE	186,097	41,128,236	7.80
ClassI/LTR/Copia	10,504	18,362,855	3.48
ClassI/LTR/Gypsy	15,917	6,919,781	1.31
ClassI/LTR/Unknown	10,784	6,405,144	1.21
ClassI/PLE	2,924	916,734	0.17
ClassI/SINE	3,565	3,996,977	0.76
ClassI/TRIM	2,156	2,412,481	0.46
ClassI/Unknown	894	302,205	0.06
ClassII/Crypton	994	183,971	0.03
ClassII/Helitron	2,237	187,098	0.04
ClassII/MITE	444	83,148	0.02
ClassII/Maverick	1,960	944,465	0.18
ClassII/TIR	313,322	54,927,849	10.41
ClassII/Unknown	18,873	2,417,365	0.46
Potential Host Gene	7,014	1,588,086	0.30
SSR	566	165,218	0.03
Unknown	75,083	23,169,180	4.39
Total	984,499	207,239,579	39.29

Supplementary Table 8. Statistics of TEs in *H. zosterae* genome.

Type	Number	Length (bp)	Rate (%)
ClassI/DIRS	1,525	1,872,980	0.41
ClassI/LINE	18,502	32,829,699	7.16
ClassI/LTR	1,549	2,654,203	0.58
ClassI/LTR/Copia	888	753,632	0.16
ClassI/LTR/Gypsy	4,163	6,530,675	1.43
ClassI/PLE|LARD	14,252	16,487,238	3.60
ClassI/SINE	364	157,570	0.03
ClassI/TRIM	2,766	3,012,874	0.66
ClassI/Unknown	347	35,280	0.01
ClassII/Crypton	1,514	605,378	0.13
ClassII/Helitron	4,085	481,059	0.10
ClassII/MITE	2,695	575,133	0.13
ClassII/Maverick	1,173	82,388	0.02
ClassII/TIR	168,736	68,196,424	14.88
ClassII/TIR|Maverick	72	104,350	0.02
ClassII/Unknown	13,155	1,387,441	0.30
Potential Host Gene	6,876	1,075,971	0.23
SSR	777	244,422	0.05
Unknown	21,933	6,681,439	1.46
Total	265,372	127,247,841	27.77

Supplementary Table 9. Statistics of the gene prediction in *H. mohnikei* and *H. zosterae*.

	Method	Software	Species	Gene Number
H. zosterae	*Ab initio*	Genscan	-	26,129
		Augustus	-	27,922
		GlimmerHMM	-	48,689
		GeneID	-	41,216
		SNAP	-	32,096
	Homology-based	GeMoMa	*Danio rerio*	18,488
			Oryzias latipes	18,543
			Salmo salar	20,404
			Hippocampus comes	19,456
	RNAseq	PASA	-	13,699
		GeneMarkS-T	-	37,548
		TransDecoder	-	52,195
	Intergration	EVM	-	21,605
H. mohnikei	*Ab initio*	Genscan	-	24,330
		Augustus	-	19,523
		GlimmerHMM	-	58,811
		GeneID	-	40,753
		SNAP	-	38,803
	Homology-based	GeMoMa	*Hippocampus zosterae*	22,585
			Syngnathus scovelli	20,132
			Hippocampus comes	20,594
			Danio rerio	19,798
	Intergration	EVM	-	21,386

Supplementary Table 10. Statistics of functional annotation of protein-coding genes in *H. zosterae*.

Annotation database	Annotated number	Percentage (%)
GO	10,542	48.79
KEGG	12,126	56.13
KOG	14,570	67.44
Pfam	18,456	85.42
Swissprot	14,617	67.66
TrEMBL	19,945	92.32
Nr	20,037	92.74
Nt	20,020	92.66
All Annotated	20,760	96.09

Supplementary Table 11. Statistics of functional annotation of protein-coding genes in *H. mohnikei*.

Annotation database	Annotated number	Percentage (%)
GO	10,796	50.48
KEGG	13,312	62.25
KOG	14,783	69.12
TrEMBL	20,288	94.87
Nr	20,379	95.29
All Annotated	20,408	95.43

Supplementary Table 12. Statistics of the non-coding RNA and pseudogenes annotation.

	RNA classification	Number	Family
H. zosterae	miRNA	642	80
	rRNA	261	4
	tRNA	2,584	25
H. mohnikei	miRNA	542	79
	rRNA	3,941	4
	tRNA	3,415	25
	**Pseudogene Number**	**Total length (bp)**	**Average length (bp)**
H. zosterae	723	3,976,796	5,500.4
H. mohnikei	1,240	5,144,777	4,149.0

Supplementary Table 13. KEGG enrichment of contracted gene families in seahorse species (Q<0.01). The statistical tests were two-sided and the FDR adjustments were made for multiple comparisons.

Pathway ID	Pathway	Count	*P*-value	*Q*-value
ko05320	Autoimmune thyroid disease	287	3.35E-208	9.60E-206
ko05330	Allograft rejection	268	9.66E-195	1.39E-192
ko05416	Viral myocarditis	280	5.49E-176	5.25E-174
ko05322	Systemic lupus erythematosus	266	9.22E-168	6.61E-166
ko05332	Graft-versus-host disease	198	2.70E-137	1.55E-135
ko04145	Phagosome	330	3.25E-125	1.56E-123
ko04940	Type I diabetes mellitus	198	1.17E-119	4.79E-118
ko05150	Staphylococcus aureus infection	210	8.49E-115	3.04E-113
ko05310	Asthma	141	6.46E-108	2.06E-106
ko04514	Cell adhesion molecules	360	4.22E-103	1.21E-101
ko05168	Herpes simplex infection	452	6.10E-102	1.59E-100
ko04672	Intestinal immune network for IgA production	168	4.24E-90	1.01E-88
ko04740	Olfactory transduction	180	2.98E-89	6.58E-88
ko04613	Neutrophil extracellular trap formation	221	8.63E-76	1.77E-74
ko05140	Leishmaniasis	185	2.32E-74	4.43E-73
ko05152	Tuberculosis	262	1.59E-65	2.85E-64
ko04612	Antigen processing and presentation	159	8.82E-64	1.49E-62
ko04640	Hematopoietic cell lineage	188	2.14E-63	3.42E-62
ko05323	Rheumatoid arthritis	183	1.02E-56	1.54E-55
ko05169	Epstein-Barr virus infection	274	2.61E-53	3.74E-52
ko04650	Natural killer cell mediated cytotoxicity	163	8.91E-50	1.22E-48

ko05130	Pathogenic Escherichia coli infection	272	3.13E-43	4.08E-42
ko05164	Influenza A	203	3.68E-40	4.60E-39
ko05414	Dilated cardiomyopathy	163	1.88E-36	2.25E-35
ko04061	Viral protein interaction with cytokine and cytokine receptor	132	2.75E-36	3.15E-35
ko05146	Amoebiasis	131	2.98E-32	3.29E-31
ko05143	African trypanosomiasis	81	9.77E-31	1.04E-29
ko04064	NF-kappa B signaling pathway	149	4.32E-29	4.43E-28
ko05135	Yersinia infection	172	1.14E-27	1.13E-26
ko04662	B cell receptor signaling pathway	109	7.45E-25	7.13E-24
ko04623	Cytosolic DNA-sensing pathway	83	3.33E-24	3.09E-23
ko05340	Primary immunodeficiency	70	2.07E-21	1.86E-20
ko05134	Legionellosis	85	3.81E-20	3.32E-19
ko04217	Necroptosis	133	6.21E-19	5.24E-18
ko05133	Pertussis	94	6.80E-19	5.58E-18
ko04666	Fc gamma R-mediated phagocytosis	115	9.85E-19	7.85E-18
ko05202	Transcriptional misregulation in cancer	187	1.35E-18	1.05E-17
ko04664	Fc epsilon RI signaling pathway	80	2.74E-18	2.07E-17
ko04625	C-type lectin receptor signaling pathway	123	2.02E-17	1.48E-16
ko04621	NOD-like receptor signaling pathway	147	5.17E-17	3.71E-16
ko05171	Coronavirus disease - COVID-19	185	1.64E-16	1.15E-15
ko00430	Taurine and hypotaurine metabolism	41	5.25E-16	3.59E-15

ko00603	Glycosphingolipid biosynthesis - globo and isoglobo series	40	1.46E-15	9.72E-15
ko00590	Arachidonic acid metabolism	71	2.60E-15	1.70E-14
ko05321	Inflammatory bowel disease	81	4.67E-15	2.98E-14
ko05410	Hypertrophic cardiomyopathy	103	2.50E-13	1.56E-12
ko00601	Glycosphingolipid biosynthesis - lacto and neolacto series	50	3.17E-13	1.94E-12
ko00140	Steroid hormone biosynthesis	60	3.06E-12	1.83E-11
ko04974	Protein digestion and absorption	95	2.45E-11	1.44E-10
ko04260	Cardiac muscle contraction	83	1.19E-10	6.84E-10
ko00480	Glutathione metabolism	60	1.23E-08	6.94E-08
ko05417	Lipid and atherosclerosis	152	2.33E-08	1.28E-07
ko04972	Pancreatic secretion	82	2.54E-08	1.37E-07
ko04610	Complement and coagulation cascades	64	2.78E-08	1.48E-07
ko05034	Alcoholism	100	3.56E-08	1.86E-07
ko00515	Mannose type O-glycan biosynthesis	40	3.72E-08	1.91E-07
ko04210	Apoptosis	125	3.95E-08	1.99E-07
ko05163	Human cytomegalovirus infection	163	8.79E-08	4.35E-07
ko05166	Human T-cell leukemia virus 1 infection	165	9.39E-08	4.57E-07
ko05412	Arrhythmogenic right ventricular cardiomyopathy	80	1.13E-07	5.41E-07
ko00130	Ubiquinone and other terpenoid-quinone biosynthesis	20	1.65E-07	7.78E-07

ko04072	Phospholipase D signaling pathway	119	2.97E-07	1.37E-06
ko05145	Toxoplasmosis	93	3.28E-07	1.49E-06
ko04062	Chemokine signaling pathway	122	3.87E-07	1.73E-06
ko04658	Th1 and Th2 cell differentiation	81	4.86E-07	2.15E-06
ko04020	Calcium signaling pathway	181	2.74E-06	1.19E-05
ko00591	Linoleic acid metabolism	26	2.94E-06	1.26E-05
ko05170	Human immunodeficiency virus 1 infection	141	4.56E-06	1.93E-05
ko04620	Toll-like receptor signaling pathway	76	4.76E-06	1.98E-05
ko00780	Biotin metabolism	10	4.86E-06	1.99E-05
ko05160	Hepatitis C	118	6.58E-06	2.66E-05
ko04670	Leukocyte transendothelial migration	100	1.16E-05	4.63E-05
ko04060	Cytokine-cytokine receptor interaction	152	2.01E-05	7.91E-05
ko04921	Oxytocin signaling pathway	110	2.83E-05	1.10E-04
ko05203	Viral carcinogenesis	117	6.80E-05	2.60E-04
ko04330	Notch signaling pathway	50	7.13E-05	2.69E-04
ko05167	Kaposi sarcoma-associated herpesvirus infection	128	8.18E-05	3.05E-04
ko05131	Shigellosis	150	9.12E-05	3.36E-04
ko00982	Drug metabolism - cytochrome P450	30	0.000117985	4.29E-04
ko05132	Salmonella infection	155	0.000158722	5.69E-04
ko05142	Chagas disease	78	0.000250354	8.87E-04
ko04622	RIG-I-like receptor signaling pathway	50	0.000437291	1.53E-03
ko05144	Malaria	40	0.000573867	1.98E-03

ko00400	Phenylalanine, tyrosine and tryptophan biosynthesis	10	0.000637647	2.18E-03
ko00670	One carbon pool by folate	20	0.000673374	2.27E-03
ko00061	Fatty acid biosynthesis	20	0.000758304	2.53E-03
ko04970	Salivary secretion	51	0.00099251	3.27E-03
ko00062	Fatty acid elongation	29	0.001351192	4.41E-03
ko00520	Amino sugar and nucleotide sugar metabolism	40	0.001474135	4.75E-03
ko00592	alpha-Linolenic acid metabolism	20	0.001932865	6.16E-03
ko04218	Cellular senescence	102	0.002148802	6.78E-03

Supplementary Table 14. KEGG enrichment of rapidly evolving genes (REGs) in seahorse species (Q-value<0.05). The statistical tests were two-sided and the FDR adjustments were made for multiple comparisons.

Pathway ID	Pathway	Count	*P*-value	*Q*-value
ko04110	Cell cycle	160	2.74E-41	7.51E-39
ko03050	Proteasome	60	5.40E-18	7.40E-16
ko00532	Glycosaminoglycan biosynthesis - chondroitin sulfate / dermatan sulfate	40	4.42E-14	4.04E-12
ko03020	RNA polymerase	40	1.13E-12	7.72E-11
ko04114	Oocyte meiosis	80	2.21E-11	1.21E-09
ko04934	Cushing syndrome	121	7.07E-11	3.23E-09
ko04950	Maturity onset diabetes of the young	41	9.22E-11	3.61E-09
ko04080	Neuroactive ligand-receptor interaction	250	1.08E-10	3.71E-09
ko04914	Progesterone-mediated oocyte maturation	70	1.64E-10	4.85E-09
ko00514	Other types of O-glycan biosynthesis	49	1.77E-10	4.85E-09
ko00512	Mucin type O-glycan biosynthesis	40	2.77E-10	6.89E-09
ko03060	Protein export	30	5.44E-10	1.24E-08
ko00563	Glycosylphosphatidylinositol (GPI)-anchor biosynthesis	30	1.52E-09	3.21E-08
ko04550	Signaling pathways regulating pluripotency of stem cells	100	1.94E-07	3.80E-06
ko03450	Non-homologous end-joining	20	2.69E-07	4.91E-06
ko03040	Spliceosome	80	3.80E-07	6.50E-06
ko00513	Various types of N-glycan biosynthesis	40	7.59E-07	1.22E-05
ko05217	Basal cell carcinoma	61	8.41E-07	1.28E-05

ko04120	Ubiquitin mediated proteolysis	79	1.17E-05	1.68E-04
ko00750	Vitamin B6 metabolism	10	1.67E-05	2.19E-04
ko04913	Ovarian steroidogenesis	40	1.68E-05	2.19E-04
ko00590	Arachidonic acid metabolism	40	3.97E-05	4.75E-04
ko03022	Basal transcription factors	30	4.23E-05	4.83E-04
ko04917	Prolactin signaling pathway	50	4.57E-05	5.01E-04
ko00510	N-Glycan biosynthesis	40	5.75E-05	6.06E-04
ko04150	mTOR signaling pathway	90	7.14E-05	7.25E-04
ko00591	Linoleic acid metabolism	20	9.50E-05	9.30E-04
ko04975	Fat digestion and absorption	30	0.000147	1.39E-03
ko00592	alpha-Linolenic acid metabolism	20	0.000154	1.41E-03
ko04137	Mitophagy - animal	50	0.000216	1.91E-03
ko05340	Primary immunodeficiency	30	0.00038	3.25E-03
ko04068	FoxO signaling pathway	80	0.000704	5.51E-03
ko05225	Hepatocellular carcinoma	92	0.000765	5.82E-03
ko04141	Protein processing in endoplasmic reticulum	90	0.000828	6.13E-03
ko00740	Riboflavin metabolism	10	0.001416	9.99E-03
ko05226	Gastric cancer	91	0.001422	9.99E-03
ko00640	Propanoate metabolism	20	0.002889	1.98E-02
ko05224	Breast cancer	91	0.003985	2.66E-02
ko00565	Ether lipid metabolism	30	0.004833	3.15E-02
ko00790	Folate biosynthesis	20	0.00582	3.65E-02

Supplementary Table 15. KEGG enrichment of lineage-specific mutated genes (LSGs) in seahorse species (Q-value<0.05). The statistical tests were two-sided and the FDR adjustments were made for multiple comparisons.

Pathway ID	Pathway	Count	Q-value
ko04120	Ubiquitin mediated proteolysis	220	2.54E-48
ko03040	Spliceosome	180	2.91E-32
ko00563	Glycosylphosphatidylinositol (GPI)-anchor biosynthesis	60	6.46E-23
ko04150	mTOR signaling pathway	190	1.95E-21
ko03015	mRNA surveillance pathway	110	9.20E-21
ko04914	Progesterone-mediated oocyte maturation	120	1.52E-20
ko03420	Nucleotide excision repair	80	4.72E-20
ko03022	Basal transcription factors	70	1.71E-18
ko03020	RNA polymerase	60	2.02E-17
ko04341	Hedgehog signaling pathway - fly	50	1.25E-15
ko00280	Valine, leucine and isoleucine degradation	70	1.26E-14
ko04960	Aldosterone-regulated sodium reabsorption	60	4.09E-13
ko04664	Fc epsilon RI signaling pathway	80	2.07E-12
ko04140	Autophagy - animal	160	4.72E-11
ko03013	Nucleocytoplasmic transport	100	7.75E-11
ko03060	Protein export	40	2.49E-10
ko04340	Hedgehog signaling pathway	59	1.03E-09
ko05110	Vibrio cholerae infection	60	8.08E-09
ko04966	Collecting duct acid secretion	40	9.65E-09
ko04114	Oocyte meiosis	100	1.37E-08
ko04910	Insulin signaling pathway	129	2.60E-08
ko03018	RNA degradation	80	6.18E-08
ko05017	Spinocerebellar ataxia	131	1.24E-07
ko04070	Phosphatidylinositol signaling system	100	1.59E-07
ko04919	Thyroid hormone signaling pathway	110	4.04E-07
ko03440	Homologous recombination	50	8.87E-07
ko04611	Platelet activation	110	1.30E-06
ko04662	B cell receptor signaling pathway	80	1.30E-06
ko04141	Protein processing in endoplasmic reticulum	147	1.34E-06
ko04216	Ferroptosis	50	1.68E-06

ko04213	Longevity regulating pathway - multiple species	70	2.19E-06
ko05203	Viral carcinogenesis	150	2.54E-06
ko00510	N-Glycan biosynthesis	60	5.13E-06
ko05206	MicroRNAs in cancer	149	7.55E-06
ko00900	Terpenoid backbone biosynthesis	30	9.70E-06
ko03050	Proteasome	50	1.00E-05
ko04722	Neurotrophin signaling pathway	100	1.51E-05
ko04110	Cell cycle	110	1.96E-05
ko04071	Sphingolipid signaling pathway	100	2.19E-05
ko05205	Proteoglycans in cancer	180	2.38E-05
ko04550	Signaling pathways regulating pluripotency of stem cells	130	2.69E-05
ko04917	Prolactin signaling pathway	70	7.09E-05
ko04212	Longevity regulating pathway - worm	67	1.10E-04
ko03030	DNA replication	40	1.10E-04
ko04361	Axon regeneration	80	1.27E-04
ko00071	Fatty acid degradation	40	1.90E-04
ko04013	MAPK signaling pathway - fly	70	2.71E-04
ko05417	Lipid and atherosclerosis	157	5.89E-04
ko04929	GnRH secretion	60	8.38E-04
ko04146	Peroxisome	70	8.51E-04
ko01524	Platinum drug resistance	60	9.01E-04
ko04214	Apoptosis - fly	50	9.42E-04
ko00520	Amino sugar and nucleotide sugar metabolism	50	1.05E-03
ko04750	Inflammatory mediator regulation of TRP channels	80	1.17E-03
ko00534	Glycosaminoglycan biosynthesis - heparan sulfate / heparin	30	2.26E-03
ko05016	Huntington disease	190	3.31E-03
ko03010	Ribosome	90	4.20E-03
ko04360	Axon guidance	121	4.88E-03
ko04666	Fc gamma R-mediated phagocytosis	80	5.85E-03
ko04068	FoxO signaling pathway	110	5.95E-03
ko04660	T cell receptor signaling pathway	80	5.95E-03
ko04152	AMPK signaling pathway	90	9.31E-03
ko04218	Cellular senescence	120	1.12E-02

ko04062	Chemokine signaling pathway	120	1.17E-02
ko00270	Cysteine and methionine metabolism	40	1.31E-02
ko00440	Phosphonate and phosphinate metabolism	10	1.34E-02
ko00513	Various types of N-glycan biosynthesis	40	1.41E-02
ko05100	Bacterial invasion of epithelial cells	60	1.62E-02
ko04392	Hippo signaling pathway - multiple species	20	1.79E-02
ko04145	Phagosome	110	1.92E-02
ko00061	Fatty acid biosynthesis	20	2.80E-02
ko00565	Ether lipid metabolism	40	3.17E-02
ko05231	Choline metabolism in cancer	80	3.57E-02
ko00512	Mucin type O-glycan biosynthesis	30	3.88E-02

Supplementary Table 16. GenBank accession numbers and gene ID for *tlx1*.

Species	Common Name	Gene	GenBank NO.
Homo sapiens	Human	*tlx1*	ENSCHIG00000017143
Mus musculus	Mouse	*tlx1*	ENSECAG00000013342
Capra aegagrus	Goat	*tlx1*	ENSPCIG00000031947
Equus caballus	Horse	*tlx1*	ENSPLOG00000005518
Phascolarctos cinereus	Koala	*tlx1*	ENSGALG00000007831
Panthera leo	Lion	*tlx1*	ENSTGUG00000010029
Gallus gallus	Chicken	*tlx1*	ENSACCG00020009411
Dromaius novaehollandiae	Emu	*tlx1*	ENSACAG00000011182
Amazona collaria	Yellow-billed parrot	*tlx1*	ENSNSUG00000006606
Aquila chrysaetos	Golden eagle	*tlx1*	ENSACOG00000000581
Taeniopygia guttata	Aebra finch	*tlx1*	ENSDNVG00000014707
Chrysemys picta	Painted turtle	*tlx1*	FJ176775.1
Notechis scutatus	Mainland tiger snake	*tlx1*	NM_001085747.1
Anolis carolinensis	Anole lizard	*tlx1*	ENSCPBG00000003532
Xenopus tropicalis	Frog	*tlx1*	NM_001078371.1
Latimeria chalumnae	Coelacanth	*tlx1*	XM_006007509.2
Lepisosteus oculatus	Spotted gar	*tlx1*	XM_015347419.1
Erpetoichthys calabaricus	Reedfish	*tlx1*	ENSECRG00000007650
Gasterosteus aculeatus	Stickleback	*tlx1*	ENSGACG00000003860
Epinephelus lanceolatus	Grouper	*tlx1*	XM_033612688.1
Takifugu rubripes	Fugu	*tlx1*	XM_029834808.1
Oreochromis niloticus	Tilapia	*tlx1*	XM_003456686.5
Scophthalmus maximus	Turbot	*tlx1*	ENSSMAG00000019960
Xiphophorus maculatus	Platyfish	*tlx1*	XM_023327963.1
Oryzias latipes	Medaka	*tlx1*	XM_004076909.4
Fistularia commersonii	Cornetfish	*tlx1*	EVM0001242

Hippocampus comes	Tiger tail seahorse	*tlx1*	XM_019877033.1
Hippocampus erectus	Lined seahorse	*tlx1*	H.erectus.000527
Hippocampus zosterae	Dwarf seahorse	*tlx1*	EVM0000050.1
Hippocampus mohnikei	Japanese seahorse	*tlx1*	EVM0012797.1
Esox lucius	Northern pike	*tlx1*	ENSELUG00000015373
Oncorhynchus mykiss	Rainbow trout	*tlx1a*	ENSOMYG00000003624
Oncorhynchus mykiss	Rainbow trout	*tlx1b*	ENSOMYG00000042338
Salmo salar	Atlantic salmon	*tlx1a*	ENSSSAG00000069627
Salmo salar	Atlantic salmon	*tlx1b*	ENSSSAG00000068344
Carassius auratus	Goldfish	*tlx1a*	ENSCARG00000010261
Carassius auratus	Goldfish	*tlx1b*	ENSCARG00000068195
Danio rerio	Zebrafish	*tlx1*	NM_170765.1
Clupea harengus	Herring	*tlx1*	ENSCHAG00000027734
Electrophorus electricus	Electric eel	*tlx1*	ENSEEEG00000000686
Ictalurus punctatus	Channel catfish	*tlx1*	ENSIPUG00000024339
Petromyzon marinus	Lamprey	*tlx*	XM_032950506.1
Branchiostoma floridae	Amphioxus	*tlx*	FJ176775.1
Ciona intestinalis	Ciona	*tlx*	NM_001078371.1

Supplementary Table 17. Transcriptome sequencing datasets of wild type (WT), *tlx1*$^{▲}$ (KO) and *tlx1*^{A208T} (MU) zebrafishes.

Samples ID	Fish line /Tissue	Clean reads	Clean bases	GC (%)	Q30 (%)
KO-br-1	*tlx1*$^{▲}$/brain	29,088,179	8,695,204,456	44.36%	93.80%
KO-br-2	*tlx1*$^{▲}$/brain	23,168,782	6,926,366,374	44.33%	94.21%
KO-br-3	*tlx1*$^{▲}$/brain	25,687,375	7,677,925,588	44.43%	93.61%
KO-br-4	*tlx1*$^{▲}$/brain	24,933,883	7,447,389,410	44.70%	94.22%
KO-in-1	*tlx1*$^{▲}$/intestine	26,180,712	7,827,900,066	46.71%	95.89%
KO-in-2	*tlx1*$^{▲}$/intestine	27,790,678	8,301,032,318	47.32%	96.31%
KO-in-3	*tlx1*$^{▲}$/intestine	24,569,878	7,347,019,970	46.96%	95.85%
KO-in-4	*tlx1*$^{▲}$/intestine	27,114,595	8,086,373,448	47.60%	96.07%
KO-ki-1	*tlx1*$^{▲}$/kidney	20,376,162	6,099,339,068	45.08%	95.57%
KO-ki-2	*tlx1*$^{▲}$/kidney	21,580,856	6,459,301,738	44.99%	95.28%
KO-ki-3	*tlx1*$^{▲}$/kidney	24,738,542	7,380,278,536	46.99%	95.70%
KO-ki-4	*tlx1*$^{▲}$/kidney	24,262,326	7,271,417,184	46.78%	95.00%
KO-li-1	*tlx1*$^{▲}$/liver	23,878,242	7,128,889,952	46.35%	94.66%
KO-li-2	*tlx1*$^{▲}$/liver	27,880,365	8,323,057,496	47.34%	95.05%
KO-li-3	*tlx1*$^{▲}$/liver	23,833,676	7,117,176,140	46.89%	94.72%
KO-li-4	*tlx1*$^{▲}$/liver	22,565,250	6,739,765,102	48.39%	94.70%
MU-br-1	*tlx1*^{A208T}/brain	27,571,297	8,217,544,426	44.36%	95.92%
MU-br-2	*tlx1*^{A208T}/brain	24,598,168	7,338,057,954	44.05%	95.83%
MU-br-3	*tlx1*^{A208T}/brain	29,560,399	8,829,806,410	43.58%	95.83%
MU-br-4	*tlx1*^{A208T}/brain	24,699,759	7,375,252,226	43.55%	95.60%
MU-in-1	*tlx1*^{A208T}/intestine	20,807,316	6,216,034,614	45.29%	95.93%
MU-in-2	*tlx1*^{A208T}/intestine	19,846,848	5,935,464,130	44.82%	96.03%
MU-in-3	*tlx1*^{A208T}/intestine	23,196,580	6,927,732,356	45.68%	95.79%

MU-in-4	*tlx1*^{A208T}/intestine	23,353,513	6,981,292,910	45.04%	95.66%
MU-ki-1	*tlx1*^{A208T}/kidney	21,272,731	6,361,721,340	44.77%	95.25%
MU-ki-2	*tlx1*^{A208T}/kidney	28,864,538	8,613,479,966	45.85%	95.96%
MU-ki-3	*tlx1*^{A208T}/kidney	27,275,691	8,129,697,250	46.41%	95.71%
MU-ki-4	*tlx1*^{A208T}/kidney	22,227,277	6,642,468,464	45.83%	95.72%
MU-li-1	*tlx1*^{A208T}/liver	28,269,645	8,432,017,762	46.14%	96.16%
MU-li-2	*tlx1*^{A208T}/liver	26,375,486	7,866,003,922	46.96%	96.26%
MU-li-3	*tlx1*^{A208T}/liver	27,067,092	8,060,000,754	47.22%	96.64%
MU-li-4	*tlx1*^{A208T}/liver	23,085,895	6,894,671,086	46.78%	96.48%
WT-br-1	*wild type*/brain	22,349,958	6,677,275,476	44.68%	95.84%
WT-br-2	*wild type*/brain	23,036,331	6,877,930,220	44.73%	95.67%
WT-br-3	*wild type*/brain	20,313,710	6,073,470,194	43.81%	95.33%
WT-br-4	*wild type*/brain	25,083,735	7,496,868,324	44.80%	95.43%
WT-in-1	*wild type*/intestine	22,924,105	6,846,412,620	45.03%	95.95%
WT-in-2	*wild type*/intestine	24,415,394	7,284,861,540	46.28%	96.07%
WT-in-4	*wild type*/intestine	31,204,763	9,307,471,244	46.53%	96.22%
WT-in-7	*wild type*/intestine	21,813,313	6,525,605,248	45.51%	96.11%
WT-ki-2	*wild type*/kidney	24,952,918	7,459,816,636	45.56%	93.82%
WT-ki-3	*wild type*/kidney	21,823,644	6,535,179,260	45.67%	94.32%
WT-ki-4	*wild type*/kidney	21,840,569	6,538,230,102	47.62%	94.24%
WT-ki-7	*wild type*/kidney	19,186,928	5,728,682,916	45.14%	95.68%
WT-li-1	*wild type*/liver	23,669,604	7,067,522,896	47.06%	96.93%
WT-li-2	*wild type*/liver	22,481,236	6,721,992,850	47.49%	96.70%
WT-li-3	*wild type*/liver	19,720,245	5,906,881,770	45.89%	96.04%
WT-li-4	*wild type*/liver	21,486,047	6,430,134,868	47.64%	96.90%

Supplementary Table 18. Primers sequences used in the present study for qRT-PCR.

Gene	Primer sequence (from 5' to 3')
gata3	Forward: CAACGCTTGCGGACTCTACT
	Reverse: GCTCTTGTCCATCAGGCTCT
T-bet	Forward: GTACGTGGACGTGGTTCTGG
	Reverse: ATCATCTGCGCCACGTTGTT
cd8a	Forward: ATCCGCGAGGACTTTCTCAT
	Reverse: GTCGCGTTCTCACATGAACA
cd79a	Forward: ATCATCACGCCAGGGACCTA
	Reverse: GCTCGCTACCTTGTCAGAGT
C3.2	Forward: GCTTCCGTGTTGACGAGAGG
	Reverse: CAGCAGCGGGTTGTAAGATG
il12a	Forward: TTTGTTGAGAGGCGCTGGAA
	Reverse: GGAGATGTAGCCCATCGCTC
il12b	Forward: GTGGCAAAGAGAAATGCCGT
	Reverse: GCAAGTGTACTCTCCGAGCA
tgfb1	Forward: TGTCCACGTGCAAGACCCTA
	Reverse: CTGCTCCCTTAGCGTCTCCT
β-actin	Forward: GATCGTGCGTGACATCAAGGAG
	Reverse: CTGCTCCCTTAGCGTCTCCT

Supplementary References

1. Griffiths J.S. *et al.* Rfam: annotating non-coding RNAs in complete genomes. *Nucleic Acids Res.* **33**, 121-124 (2005).
2. Lowe, T. M. & Eddy, S. R. tRNAscan-SE: a program for improved detection of transfer RNA genes in genomic sequence. *Nucleic Acids Res.* **25**, 955-964 (1997).
3. She, R., Chu, J. S. C., Wang, K., Pei, J. & Chen, N. GenBlastA: enabling BLAST to identify homologous gene sequences. *Genome Res.* **19**, 143-149 (2009).
4. Birney, E., Clamp, M. & Durbin, R. GeneWise and genomewise. *Genome Res.* **14**, 988-995 (2004).
5. Hughes, L. C. *et al.* Comprehensive phylogeny of ray-finned fishes (Actinopterygii) based on transcriptomic and genomic data. *Proc. Natl. Acad. Sci. U.S.A.* **115**, 6249-6254 (2018).
6. Xie, C. *et al.* KOBAS 2.0: a web server for annotation and identification of enriched pathways and diseases. *Nucleic Acids Res.* **39**, 316-322 (2011).
7. Lenti, E. *et al.* Transcription factor TLX1 controls retinoic acid signaling to ensure spleen development. *J. Clin. Invest.* **126**, 2452-2464 (2016).
8. Brendolan, A. *et al.* A Pbx1-dependent genetic and transcriptional network regulates spleen ontogeny. *Development* **132**, 3113-3126 (2005).
9. Koss M*, et al.* Congenital Asplenia in Mice and Humans with Mutations in a Pbx/Nkx2-5/p15 Module. *Dev Cell* **22**, 913-926 (2012).
10. Xie L, Tao YX, Wu RH, Ye Q, Xu H, Li Y. Congenital asplenia due to a tlx1 mutation reduces resistance to Aeromonas hydrophila infection in zebrafish. *Fish Shellfish Immun* **95**, 538-545 (2019).
11. Kanzler, B. & Dear, T. N. Hox11 acts cell autonomously in spleen development and its absence results in altered cell fate of mesenchymal spleen precursors. *Dev. Biol.* **234**, 231-243 (2001).

12. Langenau DM*, et al.* Molecular cloning and developmental expression of Tlx (Hox11) genes in zebrafish (*Danio rerio*). *Mech Dev* **117**, 243-248 (2002).
13. Logan C, Wingate RJ, McKay IJ, Lumsden A. Tlx-1 and Tlx-3 homeobox gene expression in cranial sensory ganglia and hindbrain of the chick embryo: markers of patterned connectivity. *J Neurosci* **18**, 5389-5402 (1998).
14. Roth O*, et al.* Evolution of male pregnancy associated with remodeling of canonical vertebrate immunity in seahorses and pipefishes. *Proc Natl Acad Sci U S A* **117**, 9431-9439 (2020).
15. Pedersen, G. K. *et al.* B-1a cell development in splenectomized neonatal Mice. *Front. Immunol.* **9**, 1738 (2018).
16. Schroeder HW, Cavacini L. Structure and function of immunoglobulins. *J Allergy Clin Immun* **125**, 41-52 (2010).
17. O'Neill SK*, et al.* Monophosphorylation of CD79a and CD79b ITAM motifs initiates a SHIP-1 phosphatase-mediated inhibitory signaling cascade required for B cell anergy. *Immunity* **35**, 746-756 (2011).
18. Khirwadkar, M. A. & Kher, J. R. Study of serum immunoglobulins in normal pregnancy. *Indian J. Physiol. Pharmacol.* **35**, 69-70 (1991).
19. Collins, A. B. *et al.* Complement activation in acute humoral renal allograft rejection: diagnostic significance of C4d deposits in peritubular capillaries. *J. Am. Soc. Nephrol.* **10**, 2208-2214 (1999).
20. Hughes, A. L. Phylogeny of the C3/C4/C5 Complement-component gene family indicates that C5 diverged first. *Mol. Biol. Evol.* **11**, 417-425 (1994).
21. Sottrupjensen, L. *et al.* Common evolutionary origin of Alpha-2-Macroglobulin and Complement component-C3 and component-C4. *Proc. Natl. Acad. Sci. U.S.A.* **82**, 9-13 (1985).
22. Leber, A., Teles, A. & Zenclussen, A. C. Regulatory T cells and their role in pregnancy. *Am. J. Reprod. Immunol.* **63**, 445-459 (2010).

23. Kahn DA, Baltimore D. Pregnancy induces a fetal antigen-specific maternal T regulatory cell response that contributes to tolerance. *P Natl Acad Sci USA* **107**, 9299-9304 (2010).

24. Li CY*, et al.* Genome sequences reveal global dispersal routes and suggest convergent genetic adaptations in seahorse evolution. *Nat Commun* **12**, 1094 (2021).

25. Lin Q*, et al.* The seahorse genome and the evolution of its specialized morphology. *Nature* **540**, 395-399 (2016).
