# Supplementary material for: Immunogenetic losses co-occurred with seahorse male pregnancy and mutation in tlx1 accompanied functional asplenia
Source: Nat Commun. 2022 Dec 9;13:7610. doi: 10.1038/s41467-022-35338-7 (PMC9734139; doi:10.1038/s41467-022-35338-7)
Supplement: Supplementary file 3 — Description of Additional Supplementary Files [file 41467_2022_35338_MOESM3_ESM.pdf]

Description of Additional Supplementary Files

File Name: Supplementary Data 1
Description: The list of 1260 contracted gene families in seahorse species (P value<0.05)

File Name: Supplementary Data 2
Description: GO enrichment of contracted/expanded gene families in seahorse species (Q-value<0.05).
The statistical tests were two-sided and the adjustments were made for multiple comparisons.

File Name: Supplementary Data 3
Description: KEGG enrichment of gene losses in seahorse species (Q-value<0.05). The statistical tests
were two-sided and the adjustments were made for multiple comparisons.

File Name: Supplementary Data 4
Description: A full set of genes included in the positive selected genes (PSGs) analysis. The FDR adjustm
ents were made for multiple comparisons. Only genes with an FDR-corrected P value > 0.05 were consid
ered as positive selected genes (PSGs). In total, we identidied 103 positive selected genes (top 103 in th
e list) in seahorses. The “Gene ID” column represents the gene IDs of H.zosterae (Q-value>0.05).

File Name: Supplementary Data 5
Description: A full set of genes included in the rapidly evolving genes (REGs) analysis. The FDR adjustme
nts were made for multiple comparisons. Only genes with an FDR-corrected P value > 0.05 and the dN of
the seahorse lineage higher than that of the sister lineage of seahorse were considered as rapidly evolve
d. In total, we identidied 693 rapidly evolving genes in seahorses (Q-value >0.05). The “Gene ID” colum
n represents the gene IDs of H.zosterae.

File Name: Supplementary Data 6
Description: GO enrichment of rapidly evolving genes (REGs) in seahorse species (Q>0.05). The statistical
tests of FDR adjustments were made for multiple comparisons.

File Name: Supplementary Data 7
Description: 845 lineage-specific mutated genes (LSGs) including 1158 mutated sites identified in
seahorses. The “Gene ID” column represents the gene ids of H.zosterae.

File Name: Supplementary Data 8
Description: GO enrichment of lineage-specific mutated genes (LSGs) in seahorse species (Q-value<0.05).
The statistical tests were two-sided and the adjustments were made for multiple comparisons.

File Name: Supplementary Data 9
Description: Transcriptomic profiles of the S. biaculeatus spleens and H. erectus small white organ (top
5000 genes).

File Name: Supplementary Data 10
Description: Establishment of tlx1 knockout and point mutation zebrafish lines by CRISPR/Cas9-based
genome editing.

File Name: Supplementary Data 11
Description: The differentially expressed genes (DEGs) of tlx1▲, tlx1A208T and wild type zebrafish.

File Name: Supplementary Data 12
Description: Enrichment of gene ontology of DEGs of tlx1▲and tlx1A208T zebrafish (P-value <0.05).

File Name: Supplementary Data 13
Description: GenBank accession numbers and gene ID for batf, cd5, C3-5, ighv, and foxp gene families.

File Name: Supplementary Data 14
Description: The scaffold location and sequences of batf3, C3, C4 and foxp3 in the Aeoliscus strigatus and Fistularia tabacaria genomes.

File Name: Supplementary Data 15
Description: Accessions for previously published genomes used in this study.
