# Supplementary material for: Immunogenetic losses co-occurred with seahorse male pregnancy and mutation in tlx1 accompanied functional asplenia
Source: Nat Commun. 2022 Dec 9;13:7610. doi: 10.1038/s41467-022-35338-7 (PMC9734139; doi:10.1038/s41467-022-35338-7)
Supplement: Supplementary file 5 — Reporting Summary [file 41467_2022_35338_MOESM5_ESM.pdf]

nature portfolio

Corresponding author(s): Qiang Lin, Axel Meyer and Olivia Roth

Last updated by author(s): Nov 21, 2022

Reporting Summary

Nature Portfolio wishes to improve the reproducibility of the work that we publish. This form provides structure for consistency and transparency in reporting. For further information on Nature Portfolio policies, see our Editorial Policies and the Editorial Policy Checklist.

Statistics

For all statistical analyses, confirm that the following items are present in the figure legend, table legend, main text, or Methods section.

n/a	Confirmed	
☐	☒	The exact sample size (*n*) for each experimental group/condition, given as a discrete number and unit of measurement
☐	☒	A statement on whether measurements were taken from distinct samples or whether the same sample was measured repeatedly
☐	☒	The statistical test(s) used AND whether they are one- or two-sided<br>*Only common tests should be described solely by name; describe more complex techniques in the Methods section.*
☒	☐	A description of all covariates tested
☒	☐	A description of any assumptions or corrections, such as tests of normality and adjustment for multiple comparisons
☐	☒	A full description of the statistical parameters including central tendency (e.g. means) or other basic estimates (e.g. regression coefficient) AND variation (e.g. standard deviation) or associated estimates of uncertainty (e.g. confidence intervals)
☐	☒	For null hypothesis testing, the test statistic (e.g. F, t, r) with confidence intervals, effect sizes, degrees of freedom and P value noted<br>*Give P values as exact values whenever suitable.*
☒	☐	For Bayesian analysis, information on the choice of priors and Markov chain Monte Carlo settings
☒	☐	For hierarchical and complex designs, identification of the appropriate level for tests and full reporting of outcomes
☒	☐	Estimates of effect sizes (e.g. Cohen's *d*, Pearson's *r*), indicating how they were calculated

Our web collection on statistics for biologists contains articles on many of the points above.

Software and code

Policy information about availability of computer code

Data collection	The whole-genomes of Japanese seahorse H. mohnikei and the dwarf seahorse H. zosterae were newly sequenced in this study. Accession codes in NCBI database for previously published genomes used in this study were listed as below: Homo sapiens (PRJNA31257); Mus musculus (PRJNA11777); Gallus gallus (PRJNA698609); Anolis carolinensis (PRJNA60547); Xenopus laevis (PRJNA338693); Latimeria chalumnae (PRJNA56111); Lepisosteus oculatus (PRJNA221149); Danio rerio (PRJNA11776); Takifugu rubripes (PRJEB31988); Gasterosteus aculeatus (PRJNA13579); Oryzias latipes (PRJEB43089); Xiphophorus maculatus (PRJNA72525); Oreochromis niloticus (PRJNA354796); Periophthalmus magnuspinnatus (PRJNA627707); Hippocampus erectus (PRJNA613175); Hippocampus comes(PRJNA359802). The RNA-seq data of zebrafish, aliigator pipefish spleens and lined seahorse small white organ were newly sequenced in this study.
Data analysis	There's no new software developed or used in this study. All open or commercial software and parameters used in this study for the analysis of the sequencing data are available at Figshare (https://figshare.com/projects/Immunogenetic_losses_co-occurred_with_seahorse_male_pregnancy_and_mutation_in_tlx1_accompanied_functional_asplenia/153495). For Source data are provided with this paper.

For manuscripts utilizing custom algorithms or software that are central to the research but not yet described in published literature, software must be made available to editors and reviewers. We strongly encourage code deposition in a community repository (e.g. GitHub). See the Nature Portfolio guidelines for submitting code & software for further information.

Data

Policy information about availability of data

All manuscripts must include a data availability statement. This statement should provide the following information, where applicable:

- Accession codes, unique identifiers, or web links for publicly available datasets
- A description of any restrictions on data availability
- For clinical datasets or third party data, please ensure that the statement adheres to our policy

The whole-genome raw reads and assemblies of H. zosterae and H. mohnikei have been deposited in the NCBI database under accession code PRJNA797939 (https://www.ncbi.nlm.nih.gov/bioproject/PRJNA797939/). The raw reads of the RNA-seq (including Danio rerio, S. biaculeatus and H. erectus) have been deposited in the NCBI database under accession code PRJNA799842 (https://www.ncbi.nlm.nih.gov/sra/PRJNA799842). In addition, the data of the spleens phenotype and histology are available at Figshare (https://figshare.com/projects/Immunogenetic_losses_co-occurred_with_seahorse_male_pregnancy_and_mutation_in_tlx1_accompanied_functional_asplenia/153495). Source data are provided with this paper.

Field-specific reporting

Please select the one below that is the best fit for your research. If you are not sure, read the appropriate sections before making your selection.

☒ Life sciences ☐ Behavioural & social sciences ☐ Ecological, evolutionary & environmental sciences

For a reference copy of the document with all sections, see nature.com/documents/nr-reporting-summary-flat.pdf

Life sciences study design

All studies must disclose on these points even when the disclosure is negative.

Sample size	Two animals—one adult male Japanese seahorse H. mohnikei and one adult male dwarf seahorse H. zosterae—were part of the study. For splenic observation, one H. abdominalis, one H. erectus, one S. typhle, four S. biaculeatus and one Nerophis ophidion were used. In addition, 18 seahorse species DNA samples were also used in the present study. For zebrafish splenic detection, a total of 78 individuals were used to confirm the phenotype and transcriptomic analysis.
Data exclusions	No data was excluded in this study.
Replication	Two to four biological replicates, where appropriate, have been used and described in the methods section and supplementary information. And all attempts at replication were successful.
Randomization	For zebrafish phenotype detection, samples were randomly collected. Meanwhile, the individuals were fin-clipped and PCR procedure were conducted to identify the wild type, tlx1 knockout, tlx1-mutant lines.
Blinding	No blinded analysis was performed in this study. In the present study, PCR identified wild type, tlx1 knockout, tlx1-mutant zebrafishes were collected and dissected for splenic analysis.

Reporting for specific materials, systems and methods

We require information from authors about some types of materials, experimental systems and methods used in many studies. Here, indicate whether each material, system or method listed is relevant to your study. If you are not sure if a list item applies to your research, read the appropriate section before selecting a response.

Materials & experimental systems

n/a	Involved in the study	
☒	☐	Antibodies
☒	☐	Eukaryotic cell lines
☒	☐	Palaeontology and archaeology
☐	☒	Animals and other organisms
☒	☐	Human research participants
☒	☐	Clinical data
☒	☐	Dual use research of concern

Methods

n/a	Involved in the study	
☒	☐	ChIP-seq
☒	☐	Flow cytometry
☒	☐	MRI-based neuroimaging

Animals and other organisms

Policy information about studies involving animals; ARRIVE guidelines recommended for reporting animal research

Laboratory animals	Zebrafish (Danio rerio)AB strainFour tlx1 mutant strain (-122bp, 1th exon-5bp, 1th exonG622A, 2th exonG619A, 2th exon),

Laboratory animals	Both males and females are included�Adult fish�4-months old. As for seahorses, one male H. abdominalis (5 months) and one male H. erectus (5 months) were also used in the study.
Wild animals	Wild animals used in the study including: one adult male Japanese seahorse H. mohnikei and one adult male dwarf seahorse H. zosterae, one adult male S. typhle, four adult male S. biaculeatus and one adult male Nerophis ophidion.
Field-collected samples	This study did not include field-collected samples.
Ethics oversight	All animal experiments were conducted per the guidelines and approval of the respective Animal Research and Ethics Committees of South China Sea Institute of Oceanology, Chinese Academy of Sciences.

Note that full information on the approval of the study protocol must also be provided in the manuscript.
